# High-resolution transcriptional landscape of xeno-free human induced pluripotent stem cell-derived cerebellar organoids

**DOI:** 10.1038/s41598-021-91846-4

**Published:** 2021-06-21

**Authors:** Samuel Nayler, Devika Agarwal, Fabiola Curion, Rory Bowden, Esther B. E. Becker

**Affiliations:** 1 Department of Physiology, Anatomy and Genetics, University of Oxford, Oxford, OX1 3PT United Kingdom; 2 Weatherall Institute for Molecular Medicine, University of Oxford, Oxford, OX3 7BN United Kingdom; 3 Wellcome Centre for Human Genetics, University of Oxford, Oxford, OX3 7BN United Kingdom; 4 Present Address: Walter and Eliza Hall Institute of Medical Research, Parkville, Victoria 3052 Australia; 5 Present Address: Nuffield Department of Clinical Neurosciences, University of Oxford, Oxford, OX3 9DU United Kingdom

**Keywords:** Induced pluripotent stem cells, Neuronal development, Cell type diversity, Transcriptomics

## Abstract

Current protocols for producing cerebellar neurons from human pluripotent stem cells (hPSCs) often rely on animal co-culture and mostly exist as monolayers, limiting their capability to recapitulate the complex processes in the developing cerebellum. Here, we employed a robust method, without the need for mouse co-culture to generate three-dimensional cerebellar organoids from hPSCs that display hallmarks of in vivo cerebellar development. Single-cell profiling followed by comparison to human and mouse cerebellar atlases revealed the presence and maturity of transcriptionally distinct populations encompassing major cerebellar cell types. Encapsulation with Matrigel aimed to provide more physiologically-relevant conditions through recapitulation of basement-membrane signalling, influenced both growth dynamics and cellular composition of the organoids, altering developmentally relevant gene expression programmes. We identified enrichment of cerebellar disease genes in distinct cell populations in the hPSC-derived cerebellar organoids. These findings ascertain xeno-free human cerebellar organoids as a unique model to gain insight into cerebellar development and its associated disorders.

## Introduction

The cerebellum has a major role in controlling sensorimotor functions but is also increasingly implicated in higher-order cognitive functions including language, emotion and reward. Accordingly, dysfunction of the cerebellum is associated with both motor diseases such as ataxia, dystonia and tremor, but also non-motor disorders^1,2^. Specifically, abnormal cerebellar development is increasingly linked to neurodevelopmental disorders including autism spectrum disorder and intellectual disability^3^^4^. The human cerebellum is one of the first structures of the brain to differentiate and continues to develop until several years after birth. This protracted development makes the cerebellum especially vulnerable to insult. However, the molecular mechanisms underlying the physiological development of the human cerebellum and how these go awry in developmental disorders remain incompletely understood.

The cerebellum follows a highly stereotyped developmental programme to form a well-organized laminar structure, containing one of the largest and most metabolically active neuronal types (the Purkinje neuron (PN)), as well as the most abundant neuron in the brain (the granule cell (GC))^5^. During early development, the isthmic organizer at the mid-hindbrain (MHB) boundary in the dorsal region of rhombomere 1 induces the formation of the cerebellum through the expression the lineage-specific transcription factors including OTX2, GBX2, EN1/2, and PAX2^6,7^. Unique to cerebellar development, precursors arise from two distinct germinal zones in rhombomere 1; the ventricular zone (VZ) gives rise to GABAergic neurons (PNs, interneurons, GABAergic deep cerebellar nuclei (DCN) neurons), whereas all glutamatergic neurons (GCs, unipolar brush cells, glutamatergic DCN neurons) are generated at the rhombic lip (RL). The fourth ventricle, roof plate and subsequent hindbrain choroid plexus are responsible for secretion of growth factors required for development of adjacent regions of the cerebellum, including bone morphogenetic proteins (BMPs) to direct RL formation and Sonic Hedgehog (SHH) for VZ precursor differentiation^8^. Regulatory mechanisms driving cerebellar cell-fate specification have been studied in various animal models; however, observing cerebellar development in humans poses several significant challenges, including acquisition of relevant material to study. Human induced pluripotent stem cells (hiPSCs) offer a powerful alternative model system to gain insight into human cerebellar development. Various methodologies have recently been developed to differentiate hPSCs into cerebellar neurons through the addition of growth factors that reproduce early patterning events in vitro, recapitulating MHB barrier establishment and subsequent formation of polarized cerebellar tissue characteristic of RL/VZ expansion^9–14^. Current models have mainly focussed on the differentiation of hPSC-derived PNs and subsequent maturation by co-culture with mouse cells, which hampers downstream analyses and has implications for any future potential clinical use. Moreover, the potential of current models to study other developing cerebellar cell populations has not been fully explored yet.

Here, we employ a reproducible method to generate hiPSC-derived self-organising three-dimensional organoids without the need for mouse co-culture. Using single-cell RNA sequencing (scRNA-seq), we demonstrate the presence of transcriptionally distinct populations representative of the majority of neuronal cell types in the developing cerebellum and confirm their identity and maturity through comparison with murine and human cerebellar scRNA-seq data^15,16^. We also explore the molecular consequences resulting from the addition of Matrigel to developing cerebellar organoids aimed to simulate basement membrane signalling. Basement membrane ligands have a well-known role in initiating developmentally-related signalling^17^. The addition of extra cellular matrix (ECM) molecules designed to recapitulate elements of the basement membrane is a popular method for improving culture conditions for cortical organoids^18^, but has not been investigated in cerebellar culture models. Speculation exists that the basement membrane may contribute to local, epithelial-mediated mechanical stiffness which allows basal constriction of boundary cells engaging in anterior versus posterior movement^19^. Laminin-dependent basal constriction has been shown to be involved in MHB specification^20^. Here, we show that embedding in Matrigel results in altered growth dynamics of the cerebellar organoids and influences organoid composition with a biased lineage commitment towards RL. Finally, we identify cell populations in the hiPSC-derived cerebellar organoids that are enriched for disease genes associated with cerebellar disorders. Together, our study shows that hiPSC-derived cerebellar organoids provide a xeno-free culture system that is ideally suited to model human cerebellar development and its associated disorders.

## Results

### Embryoid body differentiation recapitulates the formation of the isthmic organizer followed by RL/VZ expansion

**Figure 1:**
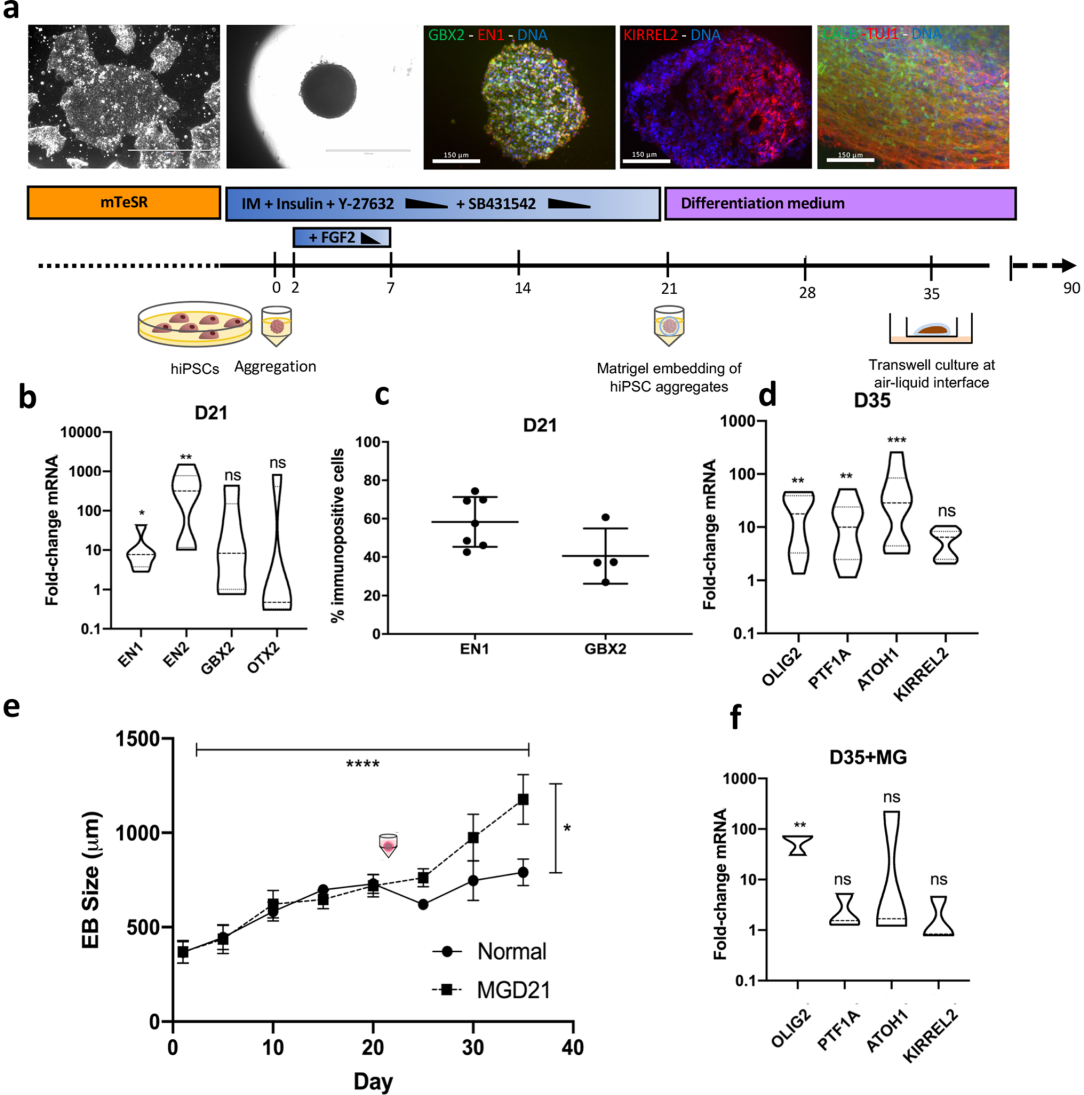
Cerebellar differentiation protocol recapitulating MHB formation and subsequent RL/VZ expansion. (**a**) Overview of cerebellar differentiation protocol. Representative images of key stages are shown above (from left to right); undifferentiated iPSCs, day 21 EBs, day 21 EB cryosections immunostained for GBX2 (green) and EN1 (red), with nuclear Hoechst staining (blue), Day 35 organoid cryosections immunostained for KIRREL2 (red), with nuclear Hoechst staining (blue), and Day 60 organoids immunostained for Calbindin (green), TUJ1 (red), with nuclear Hoechst staining (blue). Scale bars are 1000 μm (first two panels) and 150 μm (all other panels). (**b**) mRNA levels of day 21 EBs displayed as fold-change values relative to undifferentiated iPSCs. Samples were calibrated relative to *ACTB*. *EN1* (p-value 0.0346), *GBX2* (p-value 0.1147), *EN2* (p-value 0.0087) and *OTX2* (p-value 0.6429). Results are shown from 5–6 independent differentiation experiments, Mann–Whitney test. (**c**) Quantification of EN1 or GBX2 (single-channel stain) immunostaining in day 21 EB cryosections as assessed by percentage of immunoreactive nuclei divided by total nuclei. Graphs show mean with SD from 4–7 independent differentiation experiments. (**d**) Day 35 mRNA levels of *OLIG2* (p-value 0.0023), *PTF1A* (p-value 0.0072), *ATOH1* (p-value 0.0004), and *KIRREL2* (p-value 0.4211). Violin plots show median and interquartile range. Data is shown from 8–9 independent differentiation experiments tested for significance using unpaired Mann–Whitney test. (**e**) Growth rate of organoids was significant over the course of differentiation (p-value 0.0001) and significantly increased by the addition of Matrigel (MG) from day 21 onwards (p-value 0.0101) as assessed by two-way ANOVA, Dunnet multiple comparison test. Error-bars represent mean/SEM. (**f**) Matrigel encapsulation resulted in a significant increase in *OLIG2* mRNA (p-value 0.0044), but non-significant changes in *PTF1A* (p-value 0.0857), *ATOH1* (p-value 0.2527) and *KIRREL2* (p-value 0.4893). Results are shown from three differentiation experiments, unpaired Mann–Whitney test.

To generate cerebellar organoids from hiPSCs, we employed a previously established methodology aimed to mimic the self-inductive properties of the isthmic organizer^9–11^. However, instead of dissociating cultures at day 35 and subsequent co-culture with mouse progenitors, we continued the human-only cultures in a three-dimensional format at air–liquid interface (Fig. 1a). Re-aggregated hiPSCs were treated with a combination of fibroblast growth factor 2 (FGF2) and Insulin to mimic the self-inductive properties of the isthmic organizer and reproduce MHB development^9,11^. We refer to cellular aggregates as embryoid bodies (EBs) until the appearance of polarized neuroepithelial tissue and confirmation of MHB identity at day 21, from which point they are referred to as organoids. Successful acquisition of MHB fate acquisition was assessed by measuring expression of key lineage-specific transcription factors. At day 21, induction of the MHB markers *EN1* (7.66 ± 5.05-fold-change, p-value 0.0346), *EN2* (318.91 ± 183.45, p-value 0.0087), and *GBX2* (8.25 ± 34.71, p-value 0.1147) was observed, at the expense of the anterior marker *OTX2* (0.47 ± 73.66, p-value 0.6429) (Fig. 1b). Robust MHB acquisition was further confirmed by immunostaining; at day 21, 58.31 ± 12.98% cells were positive for EN1 and 40.52 ± 14.31% for GBX2 (Fig. 1a,c; Fig. 1a shows a representative dual-stained cryosection, where the majority of nuclei are immunoreactive for both markers). At day 35, acquisition of VZ/RL identity was confirmed by measurement of the VZ markers *OLIG2* (17.74 ± 7.09 fold-change, p-value 0.0023), *PTF1A* (9.96 ± 5.36, p-value 0.0072), and *KIRREL2* (6.43 ± 1.91, p-value 0.4211), and the RL marker *ATOH1* (28.53 ± 19.39, p-value 0.0004) (Fig. 1d). Consistently, immunostaining of cryosectioned organoids at 35 revealed the emergence of extensive pockets of polarized neuroepithelium showing both broad-membrane and apical accumulation of KIRREL2, one of the earliest markers of VZ precursors, and PAX6, which is expressed in RL-derivatives (Fig. 1a, Supplementary Figure S1a,b,c). Following day 60, organoids exhibited robust expression of the neuronal markers TUJ1 and Calbindin, indicating the maturation of VZ-derived neurons (Fig. 1a, Supplementary Figure S2a). The majority of Calbindin-positive cells were observed at the periphery of the organoids and exhibited bipolar morphology consistent with maturing PN progenitors (Supplementary Figure S2a). We did not observe CALBINDIN-positive cells exhibiting the characteristic elaborate morphology of mature PNs that have been described in co-culture models^9^.

### Encapsulation with Matrigel transforms growth of organoids

Matrigel has previously been used to simulate basement-membrane signalling and improve the growth of cortical organoids^18^. However, its effects on the growth of cerebellar organoids have not been investigated. To evaluate this we encapsulated EBs following early neuronal lineage commitment, as marked by appearance of polarized neuroepithelia or ‘rosettes’, in an effort to supply local factors which influence MHB development^20^. Consistent with other reports^9,10^, we observed small, round rosettes and larger ovoid-shaped structures (Supplementary Figure S2b). Given the role of basement membrane molecules in MHB formation^19,20^ we chose to embed at day 21 following the acquisition of MHB markers. Following embedding at day 21, the average diameter of embedded organoids at day 35 was 1047.37 ± 367.47 μm in contrast to 752.71 ± 168.73 μm in unencapsulated organoids, indicating that the addition of Matrigel to the culture setting results in enhanced organoid growth (p-value 0.0101) (Fig. 1e). In addition, excessive neuronal outgrowth was observed at the periphery of the organoids (Supplementary Figure S1a). The addition of Matrigel resulted in a significant increase in *OLIG2* mRNA expression (p-value 0.0044); however, it did not yield a significant difference in the expression of *PTF1A* (p-value 0.0857), *ATOH1* (p-value 0.2527) or *KIRREL2* (p-value 0.4893) (Fig. 1f). Extensive and uniform expression of KIRREL2 and PAX6 was observed in both control and embedded aggregates on day 35 of differentiation, indicating no obvious change to VZ or RL lineage-commitment upon Matrigel treatment (Supplementary Figure S1a). Upon culture of organoids past 35 days we observed necrotic or hollow pockets, possibly indicative of a lack of oxygen and/or nutrient diffusion. In order to maintain adequate oxygen supply, we continued to culture the organoids on transwell membranes. This is conventionally done with organotypic cerebellar slice cultures and was recently shown to improve neuronal survival in cortical organoids^21^.

### Individual organoid hashing and single-cell profiling of organoids reveals distinct cerebellar cell types

**Figure 2:**
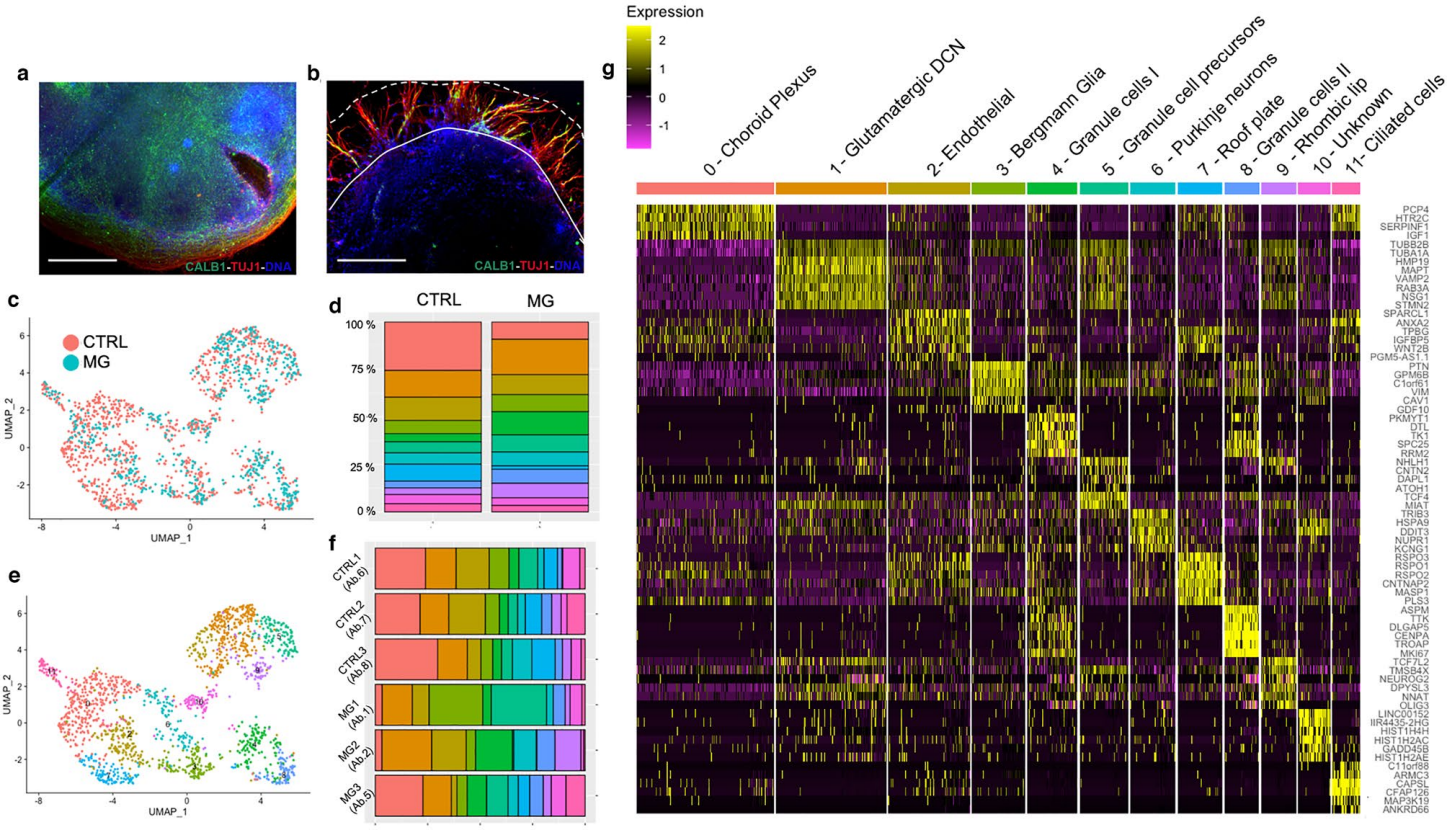
Characterisation of cerebellar organoids reveals population heterogeneity. (**a,b**) Representative images of unembedded (CTRL) organoid (**a**) and Matrigel (MG)-embedded organoid (**b**) prior to harvesting Lines demarcate the zone of Matrigel, which surrounds the organoid. Organoids immunostained for Calbindin (green) and TUJ1 (red). Nuclei were stained with Hoechst (blue). Scale bars are 500 μm. (**c**) UMAP plot after canonical correlation analysis (CCA) illustrates representation of cells across treatment conditions (pink—control, teal—MG treatment from day 21). (**d**) Visual depiction of organoid heterogeneity, colored by population, shows representation of cell types across treatment groups. Left: control (CTRL), right: MG-embedded. (**e**) UMAP plot of all cells showing 12 populations following separation after CCA at resolution 0.8. (**f**) Visual depiction of cohort heterogeneity, per individually hash-labelled organoid, colored by population, shows representation of cell types across individually barcoded organoids. Further information is given in Supplementary Figure S5. (**g**) Heatmap depicting the top six marker genes per cell population across all groups, filtered by log fold-change values. Heatmap made with Seurat (v3.2.1).

Organoids were allowed to mature for an additional 55 days at the air–liquid interface on transwell membranes before transcriptional profiling using scRNA-seq. Six organoids, each labelled with unique oligonucleotide hashtags, were sequenced as two hash-pools (2526 total cells prior to QC). Each hash-pool consisted of three control and three Matrigel-embedded organoids, harvested on day 90 of culture. Representative images are shown of organoids following culture on transwells with and without Matrigel embedding at day 21 (Fig. 2a,b). Given recent findings regarding the disparate and rapidly changing cell type-specific metabolic activity across developing neurons^22^, conventional mitochondrial gene content QC cut-offs were discarded in favour of including all singlets regressed for mitochondrial content. Mitochondrial gene expression, unique molecular identifiers (UMIs) and gene counts are shown in Supplementary Figure S3, indicating near uniform mitochondrial and gene content across samples and hash pools, respectively.

Following Louvain clustering, representation of equivalent cell populations was evident in both treatment groups following integration (Fig. 2c). Optimal clustering to distinguish between cerebellar cell types was determined by visualizing separation of related clusters in pooled data at increasing resolutions using the ClusTree package (Supplementary Figure S4) as well as by manual curation of the cluster biomarkers with canonical cerebellar markers. Following integration of treatment cohorts to identify common sources of biological variation, UMAP projection of canonical correlates at a resolution of 0.8 distinguished 12 distinct populations mutually occupying common transcriptional space (Fig. 2e,d).

Individual organoid hash-labelling allowed us to chart the population composition per organoid. The majority of populations were represented across each organoid and treatment condition (Fig. 2d,f). However, population heterogeneity per organoid was greater in samples embedded in Matrigel compared to controls (Fig. 2f, Supplementary Figure S5).

We next employed differential expression testing to identify the top common genes driving separation of common populations across integrated cohorts (Fig. 2g, Supplementary Tables S1-12). Population 0 (Fig. 2g, pink; top genes *PCP4, HTR2C, SERPINF1, IGF1*) expressed markers consistent with fourth ventricle derivatives and the hindbrain choroid plexus including *TTR, ID1, SPARC* and also *BMP7*, which is known to be actively secreted from this region^23^. Population 1 (orange; top genes *HMP19, MAPT, VAMP2, RAB3A, NSG1 & STMN2)* expressed *TCF7L2*, recently identified to mark RL-derived GCs and glutamatergic DCN^24^. This population also expressed *LHX9*, a marker of ventral RL-DCN, and *DCX*, which is expressed as DCN neurons undertake migration to the nuclear transitory zone^25^. Population 2 (beige; top genes *SPARCL1, ANXA2, TPBG, IGFBP5, WNT2B, PGM5-AS1.1*) expressed endothelial, astroglial and roof plate markers including *KRT18, IGFBP5, SPARCL1* and *PLTP*. Population 3 (lime; top genes *PTN, GPM6B, C1orf61, VIM, CAV1, GDF10*) exhibited expression of the characteristic Bergmann glia marker *FABP7*. We also noted expression within this population of *PTPRZ1*, recently shown to mark Bergmann glia^15^, and also *S100β* . Population 4 (green; top genes *PKMYT1, DYL, TK1, SPC23, RRM2*) also expressed *PCNA, MKI67* and *TOP2A* consistent with proliferative GCs. Population 5 (mint; top genes *NHLH1, CNTN2, DAPL1, ATOH1, TCF4, MIAT*) expressed GC precursor markers *NEUROD2, NEUROD6, PAX3, MEIS1, DCC* and *NFIB* consistent with RL derivatives. Population 6 (teal; top genes *TRIB3, HSPA9, DDIT3, NUPR1, EIF1 & KCNG1*) expressed markers consistent with PNs including *PSAP *^26^*, CALCB*^27^*, ALDOA, FTL*, *FTH1*, and *AARS*. Population 7 (blue; top genes *RSPO3, RSPO1, RSPO2, CNTNAP2, MASP1, PLS3*) also strongly expressed the genes *SPARC, S100B, LMX1A, HES1* and *TTR*, consistent with roof plate cells. Population 8 (mauve; top genes *ASPM, TTK, DLGAP5, CENPA, TROAP, MKI67*) exhibited GC markers such as *PAX6*, *NFIB* and *TCF4* as well as *ASPM,* which sustains postnatal cerebellar neurogenesis^28^. Population 9 (purple; top genes *TCF7L2, TMSB4X, NEUROG2, DPYSL3, NNAT, OLIG3*) also expressed *NEUROD1* and *NHLH1*, consistent with cells of the RL. Population 10 (pink; top genes *LINC00152, MIR4435-2HG, HIST1H4H, HIST1H2AC, GADD45B, HIST1H2AE*) identity could not be conclusively determined, although expression of *PCP4, GRM8, TRPM3* and sub-threshold detectable levels of *PCP2* suggest this may be a second PN population. Population 11 (salmon; top genes *C11orf88, ARMC3, CAPSL, CFAP126, MAP3K19, ANKRD66*) also expressed cilia-related genes *DYNC1I2, DYNLRB2, IFT22, TMEM67, FUZ*^29,30^. Equivalent cell types speculated to represent the ciliated ependymal cells, which line the ventricular walls have been identified in other organoid models^31,32^. Expression of population-enriched markers is shown from in situ hybridisation data from the Allen Brain Atlas (ABA), where the majority of markers appeared restricted to specific cell types (Supplementary Figure S6). Moreover, a meta-comparison with reported markers from the literature provided further support for the identified cluster identities by revealing enrichment for canonical marker genes (Supplementary Figure S7, full gene lists for corresponding cell types in Supplementary Tables S1-S12). Together, our initial analysis identified that most cerebellar neuronal cell types are present in the organoids, including RL, GCPs, GCs, PNs, Bergmann glia, choroid plexus, ciliated cells, endothelial cells, roof plate and glutamatergic DCN.

Given the well-defined biology regarding cell-cycle progression in the developing cerebellum^33^, we anticipated a strong cell-cycle presentation in GC populations. Indeed, the strongest patterns of cell-cycle progression emerged in clusters ascribed in identity as GCs (Supplementary Figure S8a-d). Regression of cell-cycle did not result in appreciable differences in assignation of clusters (not shown). Consolidation of these groups as one cluster showed strong expression of canonical GC markers *PAX6, NEUROD1, TCF7L2, NFIB* and *NCAM1* (Supplementary Figure S8e), which were also observed in parental RL/GCP populations. It is likely these two distinct clusters represent distinct stages of GC maturation and migration, which have been shown to be instrumental in foliation of the cerebellum^34^.

### An unbiased classification strategy confirms assignation of cerebellar cell types

Following putative identification of cluster identities, we next sought to validate our findings using an independent and unbiased approach. For this, we employed recently published datasets from the developing mouse and human cerebellum that were also generated using the 10 × platform^15,16^ and carried out data integration based on pairwise correspondences across datasets.

**Figure 3:**
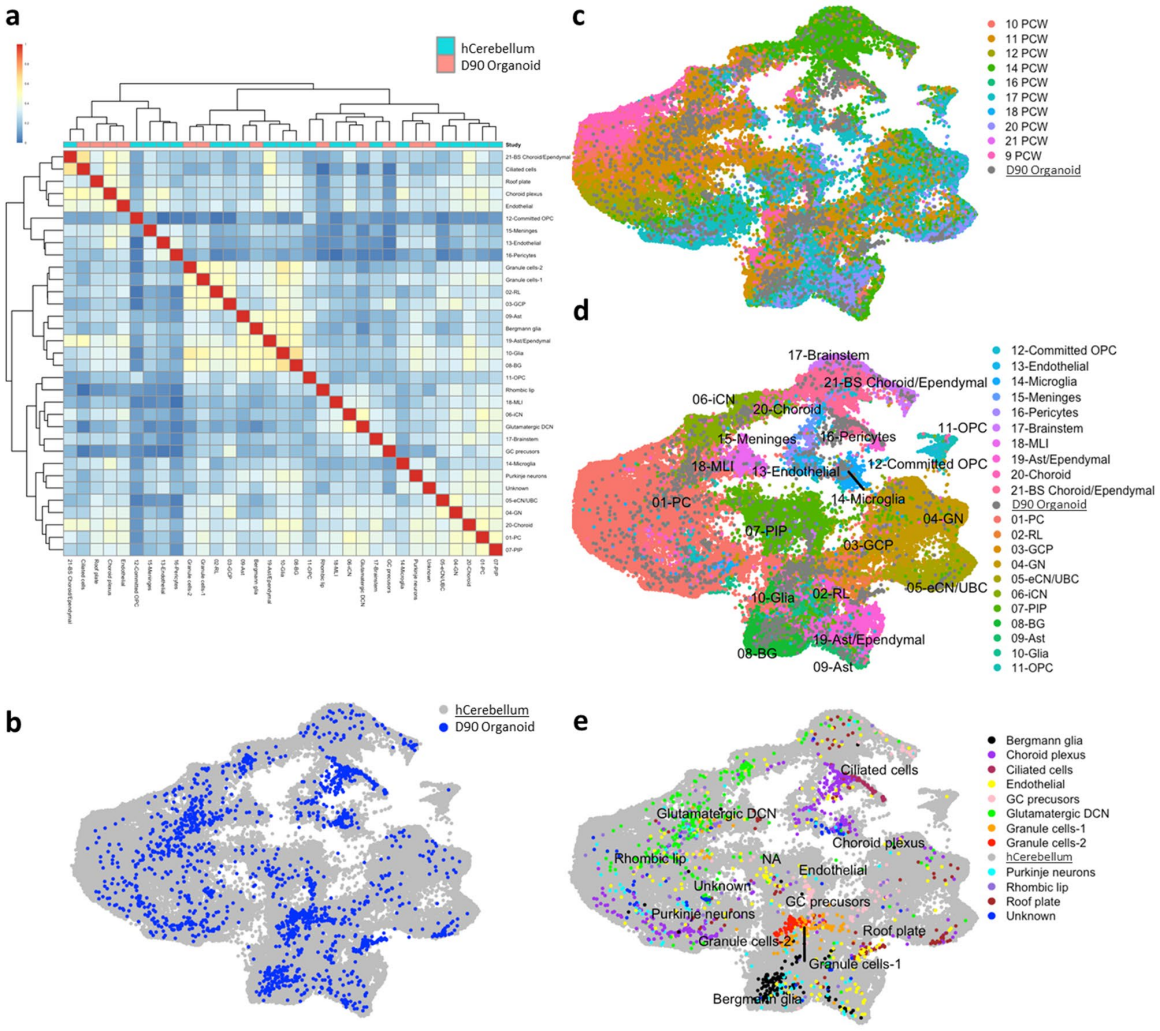
Human organoids comprise major cerebellar neuron types. (**a**) 69,174 human cells from ten developmental stages were integrated with 1653 human day 90 organoid-derived cells. Heatmap of Pearson correlation of average logged expression of highly-variable genes per cluster (cell type) from human cerebellar data^16^ against human organoids (D90), with non-parametric/ComBat batch correction, clustering by cell type. Heatmap made with Pheatmap (v1.0.12). (**b**) UMAP plot of integrated, human organoid data (blue) with human developmental data (10-21PCW, grey) shows extensive and discrete localisation to the majority of identified human developmental clusters. (**c**) UMAP plot of integrated, human organoid data color-coded according to human developmental stage (9–21 PCW) shows complex cell-type and epoch-stage clustering. Human organoid data are represented as NA entry for visualisation purposes (grey). (**d**) Integrated object highlighting human organoid cell type identity reveals close proximity of human organoid and human developmental tissue cell types. Developmental human tissue data are coloured according to identity and human organoid data are represented as NA entry (grey) for visualisation purposes. (**e**) Integrated object highlighting human developmental tissue type identity reveals close proximity of human organoid and human developmental cell types. Human organoid data are represented as coloured while tissue data is shown as NA entry (grey) for visualisation purposes. The authors’ original cell metadata labels have been preserved for consistency. Extended clustering of pseudo-bulk cluster with time course subdivisions are shown in Supplementary Figure S9.

Integration of human cerebellar organoid data with a library of 70,000 cells across human prenatal cerebellar development from 9–22 postconceptional weeks (PCW) (Fig. 3) demonstrated that the majority of cerebellar neuronal cell types that are present during early human cerebellar development are found in the hiPSC-derived cerebellar organoids at day 90 and grossly localize to their tissue counterparts, overlapping in UMAP space (Fig. 3b,d,e). Human tissue clusters represent pooled data from multiple time points (UMAP metadata labels are shown in Fig. 3d). Hierarchical clustering of average expression profiles per population showed clustering of organoid-derived ciliated cells, roof plate, choroid plexus and endothelial cells with the tissue choroid/ependymal cluster. Bergmann glia clustered proximally to tissue clusters from astrocytes, glia and Bergmann glia (Fig. 3a,e). To examine whether gene expression patterns were obfuscated by pooling of timed data, we examined average expression values of cell type clusters according to developmental age (integrated object plotted according to maturity are shown in Fig. 3c), which revealed stronger and more effective clustering once average gene expression signatures were batch-corrected and devolved to their temporal components. Primarily, we found that organoid-derived PNs clustered strongly with 20 PCW PNs (Supplementary Figure S9). Organoid-derived ciliated cells, choroid plexus, endothelial and roof plate populations all clustered most strongly with the 14PCW choroid plexus tissue population. Organoid GC populations most strongly clustered alongside 21/14PCW GC progenitors. Bergmann glia most strongly resembled 18 PCW Bergmann glia. In agreement with this, principle component analysis with Brainspan data showed clustering of pseudo-bulk organoid samples with mid-to-late prenatal samples (19–38 PCW) (Supplementary Figure S10a). To further explore this, we deconvolved bulk organoid data to generate average expression profiles from each cluster and compared these to the top 500 region-based differentially expressed genes in the Brainspan Data (p-value cutoff of < 0.05). All neuronal populations showed strongest similarity matching to the cerebellum (CB), and in particular at 16PCW (Supplementary Figure S10b). Together, these analyses show that hiPSC-derived cerebellar organoids recapitulate early processes in the developing human cerebellum with the majority of cerebellar cell types present.

Recently published 10 × scRNA-seq data obtained from the developing mouse cerebellum (embryonic day E10 to postnatal day P10) represents the most current and comprehensive atlas of cerebellar cell types across murine cerebellar development, identifying 15 distinct cell types and comprising 48 clusters across ten developmental timepoints^15^. To identify corresponding cell populations in the cerebellar organoids, we utilized a strategy to identify pairwise correspondences across murine data and project equivalent cell types across species (Supplementary Figure S11). Following one-to-one human to mouse orthologue conversion and anchor integration, UMAP projection showed clustering of human organoid and murine cerebellar cell types (Supplementary Figure S11b, d,e), showing that particular organoid-derived cells co-occupied communal transcriptional space with murine cerebellar cells. This is further illustrated by hierarchical clustering of cell type showing the highest degree of clustering across species in organoid-derived glutamatergic DCNs, ciliated cells and roof plate clusters from both studies (Supplementary Figure S11a). Organoid choroid plexus and endothelial cells clustered with the murine meninges/pia membrane cluster. While Carter et al., did not identify specifically a group of Bergmann glia, the organoid Bergmann glia cluster alongside the murine astrocyte and glia clusters. Of note, both organoid RL and GC precursor groups clustered most closely to the mouse DCN group, reflecting their close developmental origin. UMAP projection showing the total integrated population with murine cell types coloured according to temporal maturity, reveals changing representation of cell types across time (Supplementary Figure S11c). Clustering with developmental age reveals a high degree of similarity between organoid-derived human ciliated cells and murine tissue-derived ciliated cell populations at E17 (Supplementary Figure S12). Human organoid-derived roof plate clustered most strongly with the E13 murine roof plate, which also clustered proximally to E13 glia and astrocyte groups. The human organoid choroid plexus and endothelial clusters also localized proximally to these groups, indicating shared transcriptomic similarity.

Thus, using the murine and human cerebellum as a close developmental blueprint, most transcriptional signatures indicate a mixture of mid-to-late embryonic temporal maturity, suggesting that the human cerebellar organoids recapitulate discrete developmental stages of the normally developing cerebellum and constitute the majority of cerebellar cell types present during this period.

### Differential expression and trajectory reconstruction analysis reveals gross effect of Matrigel encapsulation on organoid differentiation

Following assignation of cell type clusters, we performed pseudo-time reconstruction to examine whether we could identify a developmental progression of maturing cell types. Trajectory reconstruction in pooled data showed a pattern reminiscent of the developmental cellular phylogeny of the cerebellum; cellular trajectory colour-coded by cell-type shows progression from primitive choroid plexus/roof plate cell types to RL/VZ precursors and subsequently to committed neuronal progeny bifurcating along the major glutamatergic/GABAergic lineages (Supplementary Figure S13a). Major drivers of branching included a number of recently identified markers involved in cerebellar specification^22,35^, including *WLS*, *MEIS1*, *MEIS2*, *TOP2A*, *MKI67*, *NEUROG1*, *ATOH1* and *HES3* (Supplementary Figure S13b). Pseudo-time reconstruction was largely consistent following lineage reconstruction in embedded samples (Supplementary Figure S14). Interactive pseudo-time reconstructions are available at https://plotly.com/~SamN1985/3 and https://plotly.com/~SamN1985/1 (control and Matrigel, respectively).

**Figure 4:**
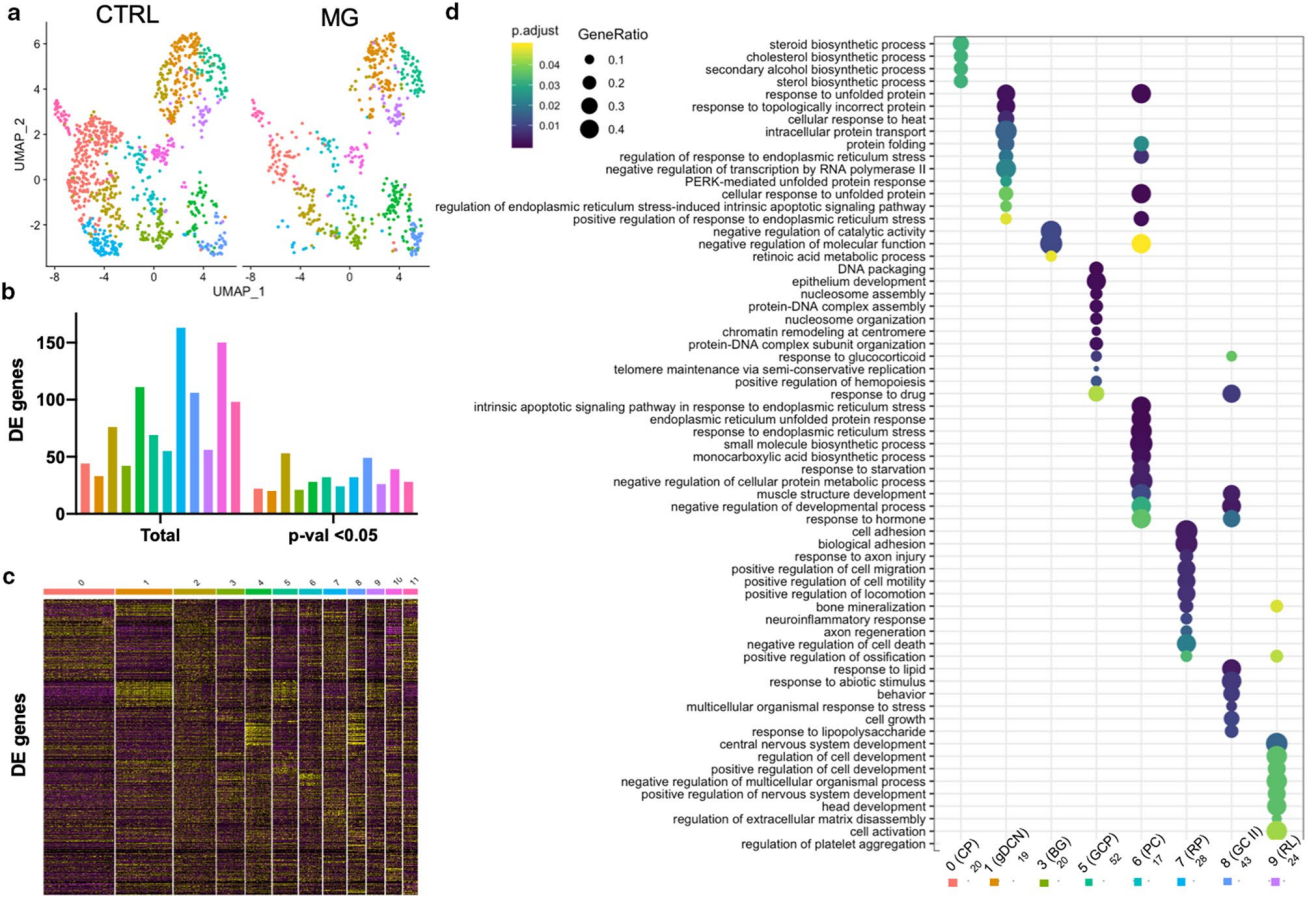
Cerebellar populations show distinct responses to Matrigel encapsulation. (**a**) UMAP projections outlining equivalent populations from control (CTRL) and Matrigel (MG)-embedded cell groups for differential-expression testing. (**b**) Graph depicting numbers of total (left) and significantly changing genes (right) per cell population. P-value < 0.05 accepted as significant. (**c**) Heatmap depicting all differentially-regulated genes between untreated and MG-embedded organoids. Heatmap made with Seurat (v3.2.1) (**d**) Dot plots showing the top differentially expressed GO pathways per cell population. The number of genes considered for each population is listed on the x-axis below cell labels.

To explore embedding-related differences, we performed differential expression testing between control and Matrigel-embedded cohorts. 46 genes were significantly differentially-regulated following Matrigel encapsulation (36 genes FDR < 0.05; 6 up, 30 down) relative to control organoids (Supplementary Figure S13c,d, Supplementary Table S16). Gene-set overrepresentation analysis returned significantly altered Gene Ontology (GO) pathways including *carboxylic acid biosynthetic process* (FDR 5.16E-05)*, organic acid biosynthetic process* (FDR 5.24E-05)*, regulation of angiogenesis* (FDR 6.21E-05)*, regulation of vasculature development* (FDR 1.1E-04)*, and tissue regeneration (*FDR 1.2E-04*).* Consistent with the transformation of growth and peripheral neuronal outgrowth following encapsulation, a number of genes concordant with survival, differentiation, and neurogenesis were among those significantly upregulated including *BIRC5*/Survivin, *HES6*, which promotes neurogenesis through the Notch pathway^36^, *DCN*, which has been shown to regulate endothelial cell–matrix interactions during angiogenesis^37^, and *PKM*, which inhibits proliferation during postnatal cerebellar neurogenesis^38^ (Fig. 4d). Furthermore, a number of hormones, chemokines and neuropeptides were amongst the genes differentially regulated with Matrigel treatment. These include *TRH*, a hormone acting on receptors normally expressed in GCs and molecular layer interneurons^39^, *IGF1,* which is known to have pleiotropic effects in all neural cells including neurons, oligodendrocytes and astrocytes^40^, *CXCL14*, a chemokine secreted by PNs directing GC migration^41^, and *CRABP2*, which regulates RA production^42^. In addition, several cell cycle-related genes were amongst the list of those differentially expressed (*CENPF, CCNB1, CCNB2, BIRC5, DCN*), suggesting that Matrigel encapsulation transforms organoids through modulation of cell cycle-dependent pathways. Furthermore, cell type-specific markers *TTR, ID1, DCN, PCP4, CXCL14, RSPO2* and *HES6* were differentially regulated following encapsulation. *ID1* (downregulated in Matrigel-embedded organoids, p-value 0.04755947) is transcriptionally active in embryonic ventricular and subependymal zones as well as being involved with postnatal development^43^. *ID* family members are known to regulate differentiation and their presence supports the detection of heterogeneity across treatment conditions. *TTR* and *PCP4* distinguish VZ precursors, while *DCN* is normally restricted to RL derivatives.

Following several independent lines of cluster identity confirmation and marker enrichment, we observed a consistent bias in population composition in the Matrigel-encapsulated samples, towards cell type identities arising from the RL (Fig. 2f & Supplementary Figure S5). To more clearly illustrate the relative contribution of RL/VZ derivatives, we performed retrospective re-clustering, distinguishing clusters comprising RL/EGL/VZ populations (Supplementary Figure S15a,b,c). Control organoids comprised 68.99 ± 0.29% VZ derivatives compared with Matrigel-embedded organoids, which showed 46.97 ± 10.43% VZ derivatives (Supplementary Figure S15d), indicating a considerable bias away from the VZ following embedding. Chi-square testing revealed consistent VZ/RL bias following treatment at both clustering resolutions (Supplementary Figures S16 and S17). Validation of RL/EGL/VZ clustering is shown by enrichment of genes driving separation at this resolution (Supplementary Figure S15b), top markers (Supplementary Figure S15c) and genes distinguishing populations (Supplementary Tables S13-15). This includes *TRPM3*, *PCP4*, *SOX2*, *TTR* and *ID1* (VZ), *NCAM1*, *TCF7L2*, *NHLH1*, *NEUROD6* (RL) and *TOP2A*, *MKI67*, *PCNA*, FOXM1 (EGL). The EGL cluster also expressed *DLGAP5*, *CDCA8*, *KIF2C*, *CDC20*, and *AURKA*. In summary, significant lineage-bias towards RL derivatives was observed upon Matrigel embedding, which was confirmed by iterated clustering at multiple resolutions.

### Population-level differential expression testing reveals cell-type specific responses to Matrigel

To evaluate the effect that Matrigel encapsulation had on specific cerebellar populations (Fig. 4a), we performed gene over-representation tests on differentially expressed genes (Fig. 4b,c, FDR < 0.05) across treatment conditions in equivalent populations. A list of differentially regulated genes is provided for each population comparison in Supplementary Tables S17-S28. GO-enriched pathways (Fig. 4d) were examined for each population following treatment. Population 0 (choroid plexus) following Matrigel encapsulation exhibited significantly altered regulation of *‘Steroid biosynthetic process’* (FDR 7.44E-05) and *‘Cholesterol biosynthetic process*’ (FDR 7.44E-05). Notably, cholesterol release from epithelial cells of the choroid plexus epithelia has been described to play an important role in cholesterol homeostasis in the cerebrospinal fluid^44^. Population 3 (Bergmann glia) showed significant changes in ‘*Retinoic acid metabolic process’ (*FDR 1.68E-04). Release of retinoic acid by radial glial cells has shown to be instrumental for numerous developmental processes including formation of the blood–brain barrier^45^, while cholesterol has been demonstrated to be vital for maturation of brain-associated microglia^46^. Population 4 (GCs-1) showed differential regulation of ‘*DNA packaging’* (FDR 9.27E-08), ‘*nucleosome assembly’* (FDR 2.00E-06), *‘chromatin remodelling at centromere’* (FDR 1.25E-05) and ‘*telomere maintenance *via* semi-conservative replication’* (FDR 1.78E-04). Population 6 (PNs) displayed enrichment for pathways ‘*Intrinsic apoptotic signalling pathway in response to endoplasmic reticulum stress’* (FDR 5.68E-09), *‘response to starvation’* (FDR 5.03E-05) and *‘monocarboxylic acid biosynthetic process’* (FDR 1.71E-05). PNs express high levels of monocarboxylate transporter 2, an integral component of the lactate transport system required for energy metabolism and neuronal activity^47^. Overall, the relative effect of Matrigel encapsulation resulted in distinct responses in the various cerebellar populations, which could be related to their function in vivo. This was most notable in RL derivatives, where encapsulation seemed to strongly influence cell cycle dynamics, driving expansion of the GC population.

### Subclustering of Purkinje neuron clusters

The majority of organoid-derived cerebellar cell types showed enrichment of canonical marker genes (Fig. 2g, Supplementary Figure S7), as well as resolution with their tissue counterparts when projected in mutual transcriptional space (Fig. 3, Supplementary Figure S9). PNs only clustered following deconvolution to their temporal components (PCA loadings are shown in Supplementary Figure S18, S19); however, we noticed a surprising lack of canonically-ascribed PN markers including *ALDOC, SKOR2, RORA* and *PCP2* localizing to the cluster we assigned PN identity. This population clustered diffusely within the human tissue PN cluster following integration (Supplementary Figure S20a shows UMAP projection with only human tissue PNs and organoid PNs projected), which was unsurprising given this cluster spanned ten developmental timepoints, presumably representing a rich biological complexity. The lack of obvious enrichment of biomarkers in our PN population may have been for a number of reasons including low cell numbers relative to tissue datasets (107 cells versus 25,724), high levels of ribosomal and stress-response genes, or intrinsic culture versus native tissue differences. One other possibility is that our clustering failed to resolve PNs at these parameters at a resolution that serviced delineation of the other cerebellar cell types because of differences in transcriptional complexity. To examine the contribution that our clustering parameters had on enrichment of PN markers, we subclustered these populations and examined overlap of enrichment of biomarkers in human and murine tissue data. We used Venice, a recent algorithm designed to overcome some of the challenges with mixed cell states/rare populations being represented by mean gene expression values^48^. BioTuring BBrowser^49^ was used to identify markers in tissue-derived PNs (pooled time points) and organoid-derived PNs, identifying 10.2% shared marker expression (977 genes logFC > 0.001, Supplementary Table S33, Supplementary Figure S20b). The ‘Unknown’ cluster, which previously clustered alongside human tissue PNs (Supplementary Figure S9), also showed diffuse clustering and overlap with more than one human tissue PN region (Supplementary Figure S20c). This cluster shared a high degree of marker overlap with PN population (63.6%), with 8.1% of biomarkers shared between the two organoid-derived populations and the tissue PN group (852 genes, Supplementary Figure S20d and Supplementary Table S34). Lastly, we compared PN markers from human tissue, murine tissue and both the PN and Unknown clusters, identifying 236 genes in common (2.2%, Supplementary Figure S20e and Supplementary Table S35). 21 of these were represented in the Ma’ayan PN gene set^50^, including *DCX* (previously observed in PNs ^51,52^), the Reelin receptor *VLDLR*, known to be involved with patterning of PN topography^53^ , *DLGAP4* (known to be expressed by PNs ^54,55^), *CRMP1* (required for PN migration^56^) as well as *RAB3C* and TNFRSF21. *NOVA2*, which regulates neuronal migration in PNs^57^ was also detected. *SOX4*, enriched here, was recently demonstrated to be expressed in PNs^16^. *CALB1* and *PCP4* were detected as biomarkers in all four groups adding further evidence for this cluster as PNs.

One possibility regarding underrepresentation of canonical biomarkers is our clustering resolution, which delineated all other cell types. Our PN and Unknown clusters could thus in fact represent two PN subtypes, separation of which would occlude conventional biomarker identification. To explore this, we grouped these cells together, also separating cells on the periphery of the BG/PN cluster, noting 180 genes including the appearance of several markers previously observed in PNs, including *NRG1*^58^, *FOXP1*^59^ and *MEF2C*^60^, and 12 of which were in common with the Ma’ayan PN gene-set list (Supplementary Figure S20f., Supplementary Table S36).

### Relevance to cerebellar disease

**Figure 5:**
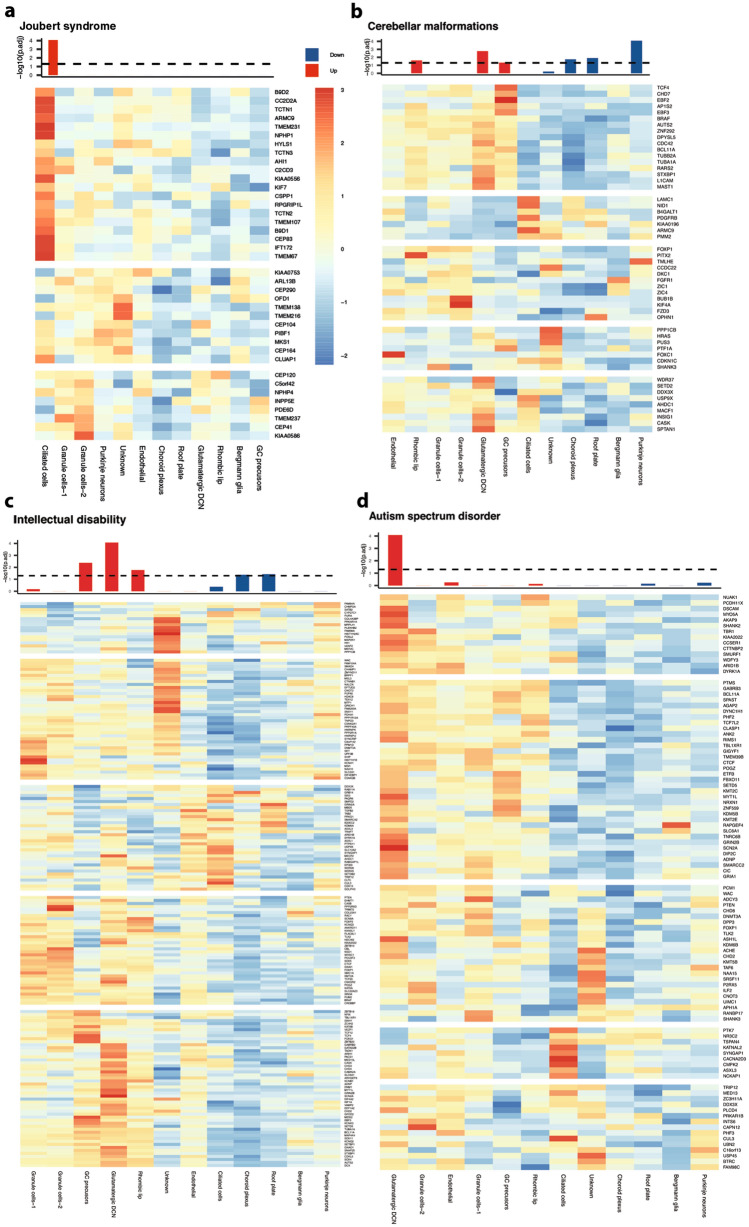
Enrichment of disease genes in discrete organoids cell populations. Heatmaps showing the average scaled expression across each of human organoid-derived cell type of risk genes for (**a**) Joubert Syndrome, (**b**) cerebellar malformation, (**c**) intellectual disability and (**d**) autism spectrum disorder. Enrichment p-values (-Log10 P value) for each cell type based on GSEA (each cell type vs all others) are shown in the top bar plots. The dashed line is the Bonferroni significance threshold (P < 0.05). Bonferroni correction was calculated by dividing nominal p-value by total number of cell types and gene sets (12 × 6). The colour of each bar in the barplot shows upregulation (red) or downregulation of gene set in particular cell type vs all other cell types based on positive or negative NES values from GSEA results (Supplementary Table S37). Heatmaps were generated using Pheatmap (v1.0.12).

The cerebellum is linked to a growing number of neurodevelopmental disorders^61^. However, the underlying genetic and molecular mechanisms remain incompletely understood. To investigate whether cerebellar organoids might serve as model systems to identify vulnerable cell populations and better understand underlying disease mechanisms in these developmental conditions, we examined transcriptomic data from individual cell clusters to identify enrichment for disease genes. We first examined genes causing Joubert syndrome^16^, a recessive neurodevelopmental ciliopathy, and identified significant positive enrichment in the ciliated cell population (Fig. 5a). We next examined the enrichment of genes that are associated with structural cerebellar malformations (cerebellar hypoplasia (CH) and Dandy-Walker syndrome (DWS))^62,63^. Genes associated with these disorders showed significant positive enrichment in RL, GC precursors and glutamatergic DCN, as well as negative enrichment in choroid plexus, roof plate, and PN populations (Fig. 5b), pointing towards potentially vulnerable cell populations in these disorders. Genes associated with intellectual disability (ID)^64–66^ showed prominent positive representation in RL, GC precursors, and glutamatergic DCN. Negative enrichment was associated with choroid plexus and roof plate clusters (Fig. 5c). Genes associated with autism spectrum disorder (ASD)^64,67–71^ were enriched in glutamatergic DCN (Fig. 5d). We did not identify any significant enrichment for genes associated with the autosomal recessive and dominant ataxias^72,73^ that represent neurodegenerative disorders of the cerebellum (Supplementary Figure S21). This is consistent with the identified developmental stage of the human cerebellar organoids at day 90, at which most ataxia genes are not yet expressed. Together, these findings underscore the utility of cerebellar organoids for studies of human neurodevelopmental disorders.

## Discussion

In this study, we have employed a robust and reproducible protocol for the generation and culture of three-dimensional cerebellar organoids from hiPSCs that does not rely on co-culture with mouse progenitors. By performing scRNA-seq of individually barcoded organoids, we demonstrate that this protocol reliably generates transcriptionally discrete populations encompassing major cerebellar neuronal cell types including RL, GC progenitors, GCs, PNs, roof plate, choroid plexus, endothelial cells, ciliated cells, Bergmann glia, and glutamatergic DCN. The identified cell populations largely correspond to cells present in the early developing human and mouse cerebellum. Moreover, we show that growth and lineage commitment of the organoids can be influenced by treatment with Matrigel. Finally, we identify cell populations in the hiPSC-derived cerebellar organoids that are enriched for disease genes associated with cerebellar developmental disorders.

Cell autonomous processes, as well as the local microenvironment drive specification and development of the cerebellum. A popular method to promote sustained growth of cortical organoids has been the addition of Matrigel to the culture setting, promoting differentiation through exposure to molecules present at the basement membrane^74^. The ECM-enriched basement membrane of the cerebellum is located underneath the pial meningeal layer, where it supplies anchor points for the endfeet of radial processes of VZ precursors, as well as acting as a barrier to migrating neurons^75,76^. These processes provide a scaffold, facilitating both cortical and cerebellar neuronal migration and correct layer formation. Evidence suggests that the basement membrane influences early patterning events at the MHB. In agreement with these observations, our data show that migratory and proliferative activity is significantly altered in Matrigel-embedded cerebellar organoids. However, Matrigel encapsulation also decreased the reproducibility of generating cerebellar cell types, with embedded organoids showing a greater degree of variability in cell type composition. Interestingly, this heterogeneity comprised a bias towards RL derivatives, including glutamatergic DCN and GCs. This is intriguing given that previous cerebellar differentiation protocols have been reliant on co-culture with isolated murine RL progenitors or GCs to provide necessary factors for growth and maturation of the VZ component^9,10,12^. Our population-level differential expression analysis suggests that GCs were especially sensitive to the addition of Matrigel, showing a greater degree of responsiveness as marked by increased numbers of significantly altered genes, particularly of those involved in cell cycle-related pathways. An alternative hypothesis involves embedding driving differential oxygen gradients and hypoxia within the organoids, a mechanism which has previously been shown to drive proliferation of GCPs in the cerebellum^77,78^. In this regard, hypoxic pockets during the differentiation may actually be advantageous.

While we showed that differences in Matrigel encapsulated organoids were subtle, encapsulation of cerebellar-patterned organoids may provide a viable alternative to costly, time-restrictive rodent RL isolations that hamper downstream analyses of differentiated human cells. However, this method should be carefully considered given batch-to-batch variability associated with Matrigel and the extra degree of handling this introduces to the culture workflow.

An exciting potential for organoid-based protocols is the fact that neighbouring regions can influence the development of adjacently located tissues. For example, both roof plate epithelium and choroid plexus epithelium are required for full development of the RL in vivo^79,80^. It has been suggested that diffusible factors such as retinoic acid influence development of the cerebellum, following secretion from the choroid plexus^81^. We show that the organoids differentiated in the absence of murine feeder layers contain choroid plexus, roof plate, ciliated cells, RL precursors, glutamatergic DCN, GCs, Bergmann glia and PNs. This suggests that organoids transit through stages akin to physiological embryonic cerebellar development, which is supported by the pseudo-temporal ordering. Future studies utilizing spatial sequencing or high-resolution imaging will give insight whether these cell types function in a manner akin to their in vivo counterparts. The presence of choroid plexus cells and endothelial cells within the organoids is particularly interesting as it raises the possibility that cerebellar organoids may possess an intrinsic angiogenic capability as well as cells of non-ectodermal origin.

In this study we did not employ SDF1 and FGF19 treatment, which encourages cerebellar plate neuroepithelium formation but is not necessary to derive mature neurons^10,14^. Future single-cell studies will be well-suited to provide molecular insights into how these growth factors promote more efficient recapitulation of the primitive cerebellar germinal zones.

Our integration with existing single-cell data from the developing mouse and human cerebellum suggests that overall, human organoids at day 90 represent mid-to-late embryonic cerebellar development. This is also reflected by the absence of some well-known, more mature markers for distinct cell populations. For example, organoid-derived PNs, which clustered most strongly with 20 PCW human tissue counterparts, failed to robustly express mature markers including *ALDOC*, *RORA,* and *PCP2*. This is consistent with the notion that PN maturation occurs during the late-gestational stage of development and is reliant on the establishment of the internal granule layer (IGL)^16^. PN maturation occurs in late gestation and is reliant on GCPs completing migration to the IGL from the EGL. In further support of this, we did not detect appreciable levels of *SHH* expression, and Calbindin-expressing neuronal cells present in D90 cerebellar organoids did not resemble the characteristic tear-drop shaped soma with a single bifurcating axon, characteristic of mature PNs. Findings in the literature suggest that PNs and GABAergic DCN arise from the VZ prior to dependence on SHH signalling^82^, consistent with the presence of immature PNs in the organoids that are still undergoing maturation, potentially in suboptimal culture settings. Moreover, organoid PNs exhibited increased expression of stress-response genes, which has previously shown to occlude identification and specification^83^. Another limitation of this study has been the limited number of identified PNs, which limited our ability to effectively survey this cluster. We have looked in greater detail at expression of genes from a number of datasets which also transcriptionally identified PNs, expanding our list of potential markers. Higher resolution studies are required to definitively place these cells on a maturation axis. Further comparison of the organoid-derived PNs to murine and human PN transcriptomes may yield some clues about supportive trophic settings that could be exploited experimentally in the future to promote survival and maturation. While we did not observe strong *SHH* expression, it is worth noting that transventricular SHH delivery, from outside of the cerebellum has been demonstrated to drive early VZ development^84^.

One limitation in comparing cell types across species and studies is incongruence of cell type labels, which are ultimately ascribed subjectively by the end-user. This, as well as technical variability may underlie the fact that not all cell types consistently clustered with their tissue counterparts. However, some cell types did not resolve with their expected counterparts, which could be down to culture-induced differences. For example, the organoid RL cluster clustered most closely with brain stem and excitatory CN. While excitatory CN arise from the RL, it is reasonable that a residual gene expression signature explains this clustering. We did not detect brainstem marker *HOXB3* and can only assume some active transcriptional programs in the brain stem resemble those in the cultured GCP/RL cells. Deconvolution of the temporal component enabled and improved cluster identification, depending on the cell type, presumably due to the transcriptional complexity/stability over time of the population. Recent advances^85,86^ to adopt standardized repositories, QC metrics and nomenclature, including and transcriptome-based taxonomic classification systems for neurons will be necessary in future work.

Our study shows that cerebellar organoid technology enables the generation of human cell types that are normally inaccessible, and provide insights into windows of development that are normally restricted. Enrichment of disease genes in distinct cell populations of the human cerebellar organoids highlights the exciting potential of this model system to provide insights into the pathogenesis of cerebellar disorders and as a valuable platform for drug screening and personalised medicine.

## Methods

### Ethics statement

The human iPSC cell line used in this study was derived from a healthy 67-year-old female donor (AHO17-3) following signed informed consent and with approval from the National Health Service, Health Research Authority, NRES Committee South Central—Berkshire, UK as previously described^95^. All experiments were performed in accordance with relevant guidelines and regulations.

### hiPSC growth and maintenance

hiPSCs from a healthy 67-year-old female donor (AHO17-3) were used as previously described^11^ between passage 25–35. Samples were tested and free of Mycoplasma using the MycoAlert kit (Lonza, Switzerland). hiPSCs were maintained on Matrigel-coated plates in a humidified CO_2_ incubator with mTeSR1 media changed daily (STEMCELL Technologies, UK). hiPSC were passaged upon reaching approximately 80% confluence using EDTA at 37 °C for three to five minutes and plated in mTESR1 with 10 μM Y-27562 (Tocris, UK). Media without Y-27562 was supplemented the day following.

### Cerebellar organoid differentiation

Figure 1a provides an overview of the differentiation protocol. Briefly, embryoid bodies (EBs) were made by dissociating sub-confluent hiPSCs with TrypLE before allowing to aggregate in ultra-low attachment 96 well-plates (PrimeSurface MS-9096 V, Sumitomo Bakelite, Japan) in growth factor-free chemically-defined medium in the presence of 7 μg/ml Insulin (Sigma-Aldrich), 50 μM Y-27562 (Sigma-Aldrich) and 10 μM SB431542 (Tocris)^9^. Approximately 1.1E04 cells were deposited into each well. Two days later, media was supplemented with 50 ng/ml FGF2 (R&D Systems). On day seven, one-third of the media was removed and supplemented with fresh media without growth factors. On day 14 full volume media replacement was performed. On day 21 of differentiation, individual EBs were embedded in undiluted Matrigel or transferred into differentiation media as free-floating aggregates (four EBs per well of a 24-well low-attachment plate). EBs were harvested on day 21 for cryosectioning/qPCR to validate acquisition of MHB fate. Medium was changed on day 28 and 35 before assessing expression of rhombic lip/ventricular zone markers. EBs were monitored for growth by photographing every two days and inspected for visual signs of differentiation including the presence of polarized neuroepithelia (rosettes). On day 35, individual EBs were transferred to transwell membranes (Millicell Cell Culture inserts, PTFE, 0.4 μm, Merck Millipore, USA) and allowed to further develop at the air–liquid interface. Organoids were harvested at multiple timepoints throughout for validation of successful cell-fate acquisition.

### Immunostaining

EBs and organoids were harvested for cryosectioning at various timepoints by fixing in 4% paraformaldehyde for 15 min at room temperature, followed by three washes of PBS. Samples were cryoprotected overnight in 20% sucrose/PBS before embedding in optimal cutting temperature compound and sectioning on a cryotome at 10 μm. Cryosections were blocked in 2% milk power/PBS for one hour at RT following permeabilization with 0.3% Triton-X100 for 10 min at RT. Sections were incubated with primary antibody diluted in blocking buffer with 0.3% Triton-X100 overnight at 4 °C followed by application of relevant fluorescent secondary-conjugated antibody (Alexa Fluor, Invitrogen). An identical preparation without the primary antibody was used as a negative control. Hoechst was applied for 5 min at RT to visualize nuclei. Immunostained sections were mounted in ProLong Gold Antifade (VECTASHIELD). Antibodies are listed in Supplementary Table S29.

### Quantitative real-time PCR

RNA was isolated from EBs/organoids on days 0, 21 and 35 using the RNeasy mini kit (Qiagen, Germany) according to manufacturer’s instructions including an on-column DNAse digestion step. cDNA was generated using the Superscript III First-strand synthesis kit (Invitrogen, USA). Quantitative real-time PCR was performed on an Applied Biosystems StepOne plus qPCR machine using Fast SYBR Green Mix according to the manufacturer’s specifications. No template and minus reverse transcriptase controls were used to test for specificity. Primers are listed in Supplementary Table S30. Relative fold-change of mRNA abundance was calculated using the ΔΔCq method. Samples were calibrated relative to *ACTB*. Results are shown from between 5 and 6 independent differentiation experiments. ΔCq values were tested for significance with P < 0.05 accepted as significant, Mann–Whitney test.

### Imaging and quantification

Imaging was performed on mounted cryosections/whole-organoids using a Zeiss upright fluorescent microscope. CellProfiler was used to segment and identify immunopositive nuclei in fluorescently-labelled cryosection images. This was divided by the total number of nuclei as ascertained by visualisation with Hoechst. Organoid growth was assessed by measuring diameter using ImageJ and assessed for significance through two-way ANOVA. Formation of polarized neuroepithelial tissue was quantified in blinded samples and subject to statistical testing using one-way ANOVA.

### Statistical analysis

Statistical tests were performed with the PRISM software package (v8) or R. qPCR data is shown from multiple independent differentiation experiments, ΔCq values tested for significance using Mann–Whitney test (two-tailed). Figure 1b; Five-six biological replicates (differentiation experiments) and three technical replicates (PCR replicates). Figure 1d; Eight-nine biological replicates (differentiation experiments), three technical replicates (PCR replicates). Figure 1e; Growth rate of organoids was assessed by two-way ANOVA (Dunnet’s multiple comparison test); Eight biological replicates (differentiation experiments), three-five technical replicates (individual organoids). F (DFn, DFd); (7, 54) = 2.466 (Interaction), (7, 54) = 17.69 (Row factor), (1, 54) = 7.110. Figure 1f; Three biological replicates (differentiation experiments) and three technical replicates (PCR replicates). Chisquare testing was used to assess lineage-specific bias in SC data at both utilized clustering resolutions. P-value < 0.05 accepted as significant for all statistical testing. Weighted Kolmogorov–Smirnov statistics and gene-set based permutations were used to derive nominal P-values for gene-set enrichment analysis for disease risk analysis.

### Preparation of organoids for single cell sequencing

Following 90 days of differentiation, three organoids embedded in Matrigel (at day 21) and three unembedded organoids were manually dissected from transwell membranes and enzymatically digested with Accumax dissociation reagent (Sigma-Aldrich) with manual trituration. Organoids were resuspended in 0.01% BSA in PBS on ice and labelled with Hash-tagged Oligonucleotide (HTO)-conjugated antibodies for B2M/ATP1B3 at RT for one hour (HTO sequences and antibodies are listed in Supplementary Table S31). Samples were processed according to the standard 10 × Chromium 3’ workflow and pooled before sequencing over two lanes (Illumina HiSeq 4000).

### Processing of single cell data

A total of 2,526 cells from two hashed pools were sequenced at an average of 53,751 reads per cell, with a median detection rate of 2,617 genes per cell. Hashed pools were demultiplexed using Seurat^87–89^ HTODemux with default options and 65% of the cells (1653) were classified as singlets. The SCTransform function in Seurat (v 3.2.1) was used to normalize UMI depth across cells using negative binomial regression followed by additional regression for mitochondrial proportion across all cells. Each hash pool was analysed separately and then also integrated and projected in UMAP space using canonical correlation analysis (CCA) and MNN (Mutual nearest neighbours). The ClusTree package and manual curation was used to visualize and determine optimal clustering parameters. Populations were identified through unsupervised clustering and projection of known cell-type specific markers using the FindConservedMarkers using default parameters^90–92^. Organoid composition was projected by plotting the fraction of each population per organoid after integration/CCA + MNN projection. Expression of cluster-specific markers was validated through comparison to sagittal ISH sections of the developing murine brain (Allen Brain Atlas). Differential expression testing was performed using the FindConservedMarkers command using a Wilcoxon Rank Sum Test using identity classes comprising the two hash pools (Matrigel-embedded samples vs control). Heatmaps were generated using Seurat (v3.2.1) and pheatmap (v1.0.12). Mouse vs human comparisons were made by performing Integration and Label Transfer^93^ and incorporating high-confidence/one-to-one human to mouse orthologues from Ensembl. Pseudo-time reconstruction was performed using Monocle. A list or identities pertaining to cluster identity from murine dataset is listed in Supplementary Table S32.

### Comparison with BrainSpan RNAseq data

Normalised gene-level expression data in RPKM for the BrainSpan RNA-seq dataset generated from post-mortem human brain were downloaded (https://www.brainspan.org/static/download.html) and only protein coding genes were retained. The human organoid SCT data was pseudobulked (Average expression) according to control and Matrigel groups and only protein-coding genes retained. A ComBat batch correction for study was performed on the log2 transformed joint BrainSpan and pseudobulked organoid protein-coding gene * sample matrix. The organoid samples were then projected onto the BrainSpan maturity PCA axis (formed by using the ComBat corrected BrainSpan data).

### Pathway analysis

GO Term Biological process over-representation tests across all cell populations was performed using the *CompareCluster* function from the Bioconductor package ClusterProfiler^94^. For each cell type population, differentially-expressed genes between treatments with a p-value < 0.05 were included in the analysis and the background universe consisted of 17,125 genes that were expressed in a minimum of 3 cells with a UMI count >  = 1.

### Cerebellar disease enrichment

We used Gene Set Enrichment Analysis (GSEA) to test enrichments of each of the six disease risk gene sets in one cell type vs all others. We calculated the average expression of each of the integrated organoid cell types and then performed GSEA with GSEA GUI app for each cell type vs all for all the 6 gene sets with the following parameters : enrichment statistic : weighted, gene ranking metric : Diff_of_Classes, Gene list sorting mode : real, Gene list ordering mode: descending, number of permutations: 10,000, permutation_type: gene labels, Max GeneSet size : 500, Minimum GeneSet size : 15, Normalisation_mode: mean and div. Nominal p-values from GSEA were corrected for multiple testing using the Bonferroni method calculated over all cell types and gene lists used [P < 0.05/(12 × 6)]. A heatmap of the average z-scores of the diseases risk gene sets across each of the cell types was plotted and the gene and cell types were clustered with the *ward.D2* method using the pheatmap R library.

## Supplementary Information

Supplementary Information 1.

Supplementary Information 2.

Supplementary Information 3.

Supplementary Information 4.

Supplementary Information 5.

Supplementary Information 6.

Supplementary Information 7.

Supplementary Information 8.

Supplementary Information 9.

Supplementary Information 10.

Supplementary Information 11.

Supplementary Information 12.

Supplementary Information 13.

Supplementary Information 14.

Supplementary Information 15.

Supplementary Information 16.

Supplementary Information 17.

Supplementary Information 18.

Supplementary Information 19.

Supplementary Information 20.

Supplementary Information 21.

Supplementary Information 22.

Supplementary Information 23.

Supplementary Information 24.

Supplementary Information 25.

Supplementary Information 26.

Supplementary Information 27.

Supplementary Information 28.

Supplementary Information 29.

Supplementary Information 30.

Supplementary Information 31.

Supplementary Information 32.

Supplementary Information 33.

Supplementary Information 34.

Supplementary Information 35.

Supplementary Information 36.

Supplementary Information 37.

Supplementary Information 38.

## Data Availability

Data is available on GEO (GSE150153).

## Code availability

Code is available on Github repository https://github.com/SamN1985/General-code and https://github.com/deevdevil88/10x_Human_Cerebellum_Organoid.
