# Supplementary material for: High-resolution transcriptional landscape of xeno-free human induced pluripotent stem cell-derived cerebellar organoids
Source: Sci Rep. 2021 Jun 21;11:12959. doi: 10.1038/s41598-021-91846-4 (PMC8217544; doi:10.1038/s41598-021-91846-4)
Supplement: Supplementary file 1 — Supplementary Information 1. [file 41598_2021_91846_MOESM1_ESM.pdf]

SUPPLEMENTARY INFORMATION

High-resolution transcriptional landscape of xeno-free human induced pluripotent stem cell-derived cerebellar organoids

Samuel Nayler[1]*, Devika Agarwal[2], Fabiola Curion[3], Rory Bowden[3,4], Esther B.E. Becker[1,5]*

[1]Department of Physiology, Anatomy and Genetics; University of Oxford; Oxford, OX1 3PT; United Kingdom

[2]Weatherall Institute for Molecular Medicine; University of Oxford; Oxford, OX3 7BN; United Kingdom

[3]Wellcome Centre for Human Genetics; University of Oxford; Oxford, OX3 7BN; United Kingdom

[4]Present address: Walter and Eliza Hall Institute of Medical Research, Parkville Victoria 3052; Australia

[5]Present address: Nuffield Department of Clinical Neurosciences; University of Oxford; Oxford, OX3 9DU; United Kingdom

*Corresponding authors: Samuel.Nayler@dpag.ox.ac.uk; Esther.Becker@ndcn.ox.ac.uk

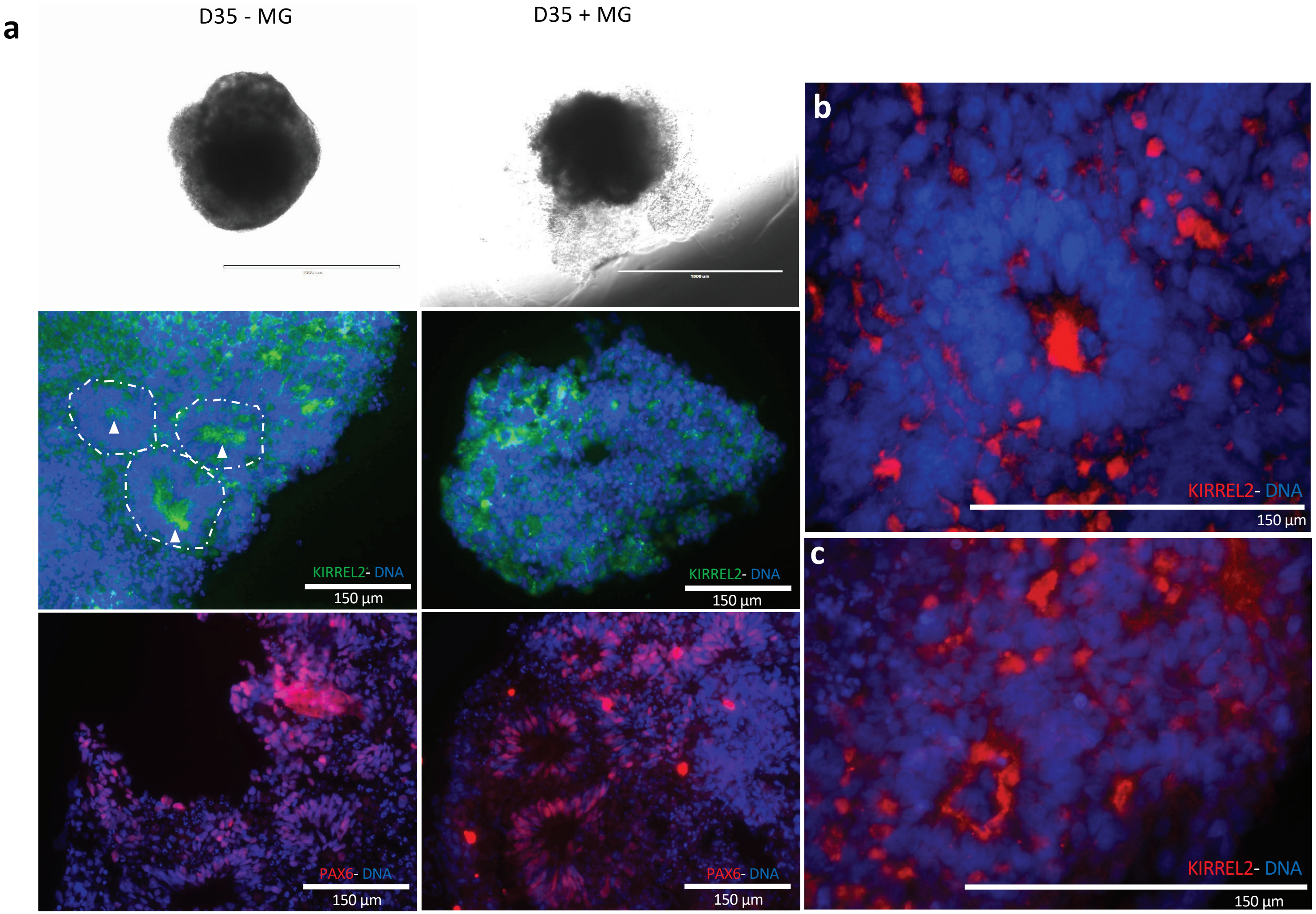

Supplementary Figure S1 – (**a**) Representative images of organoids following 35 days of differentiation without (-) and with (+) Matrigel (MG)-embedding (left & right columns respectively). Top panel shows light-microscope images. Scale bar is is 1000 µm. Middle panel shows KIRREL2 labelling (green). Scale bar is is 150 µm, with polarized neuroepithelium highlighted by a dashed line. Apical accumulation of KIRREL2 is marked by arrows. Final panel shows PAX6 labelling (red). (**b & c**) High magnification images of KIRREL2 (red) labels the membrane/lumen of neuronal rosettes in Day 35 organoids. Scale bar is 150 µm. Related to Figure 1.

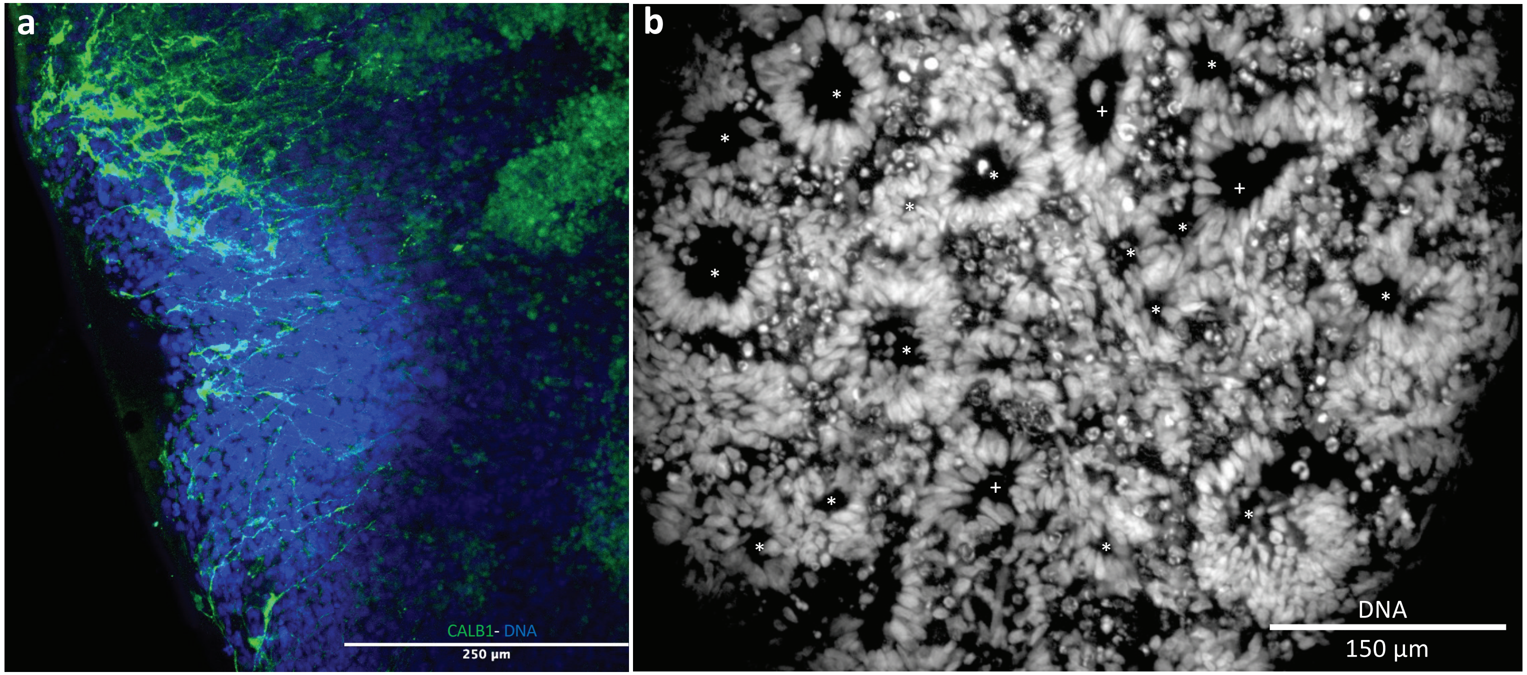

Supplementary Figure S2 – (**a**) Calbindin (green) staining of control organoids following 62 days of differentiation. Scale bar is 250 µm. Hoechst stains the nucleus. Related to Figure 1. (**b)** Representative grayscale image of organoid section at D35 showing characteristic ovoid (+) and round (*) polarised tissue. Hoechst stains the nucleus. Scale bar is 150 µm.

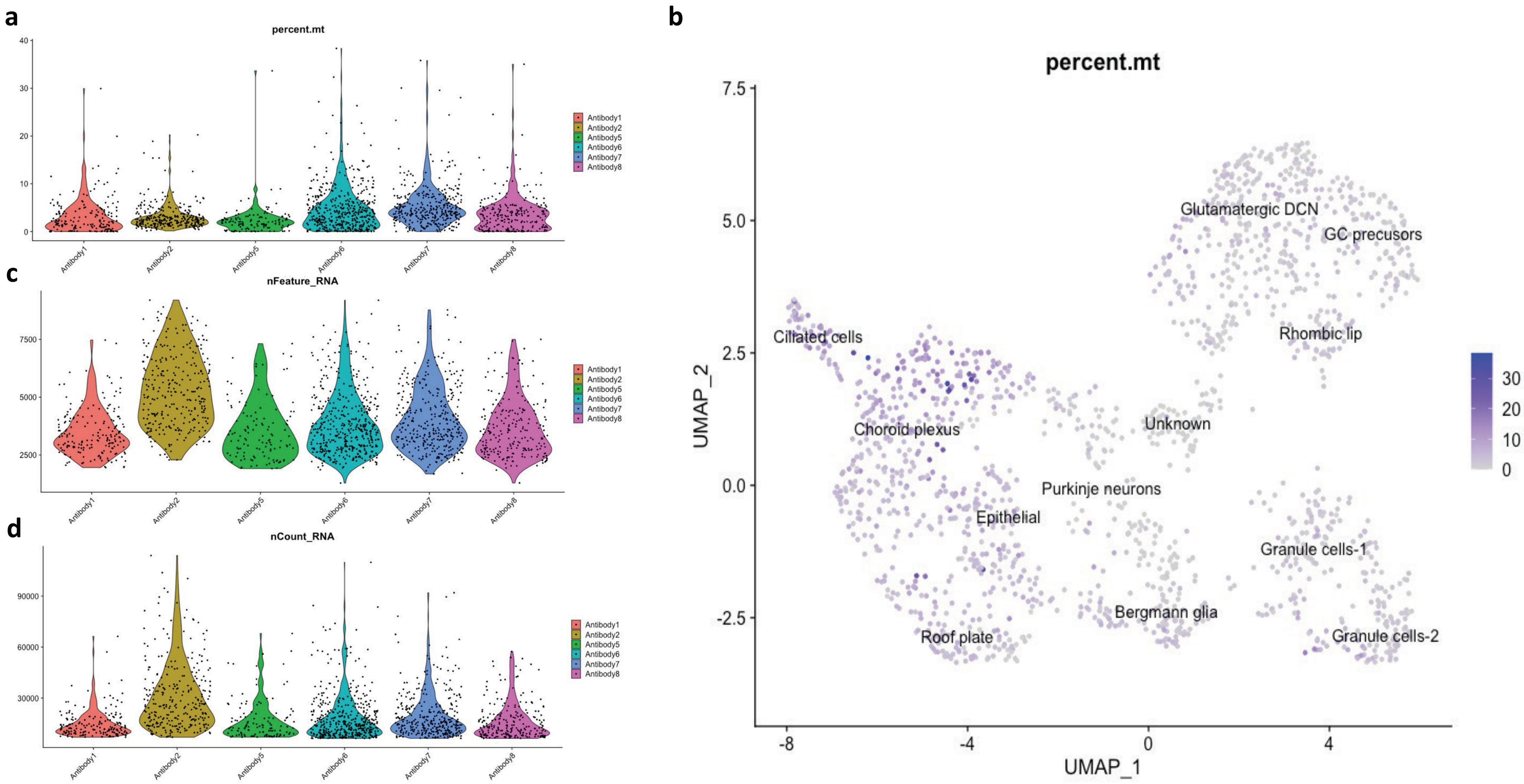

Supplementary Figure S3 – (**a**) Violin plots depicting mitochondrial gene expression in individually-barcoded organoids, (**b**) Pooled mitochondrial values projected in UMAP space. (**c**) Violin plot depicting gene content in all organoids and (**d**) UMI distribution in all organoids. Related to Figure 2.

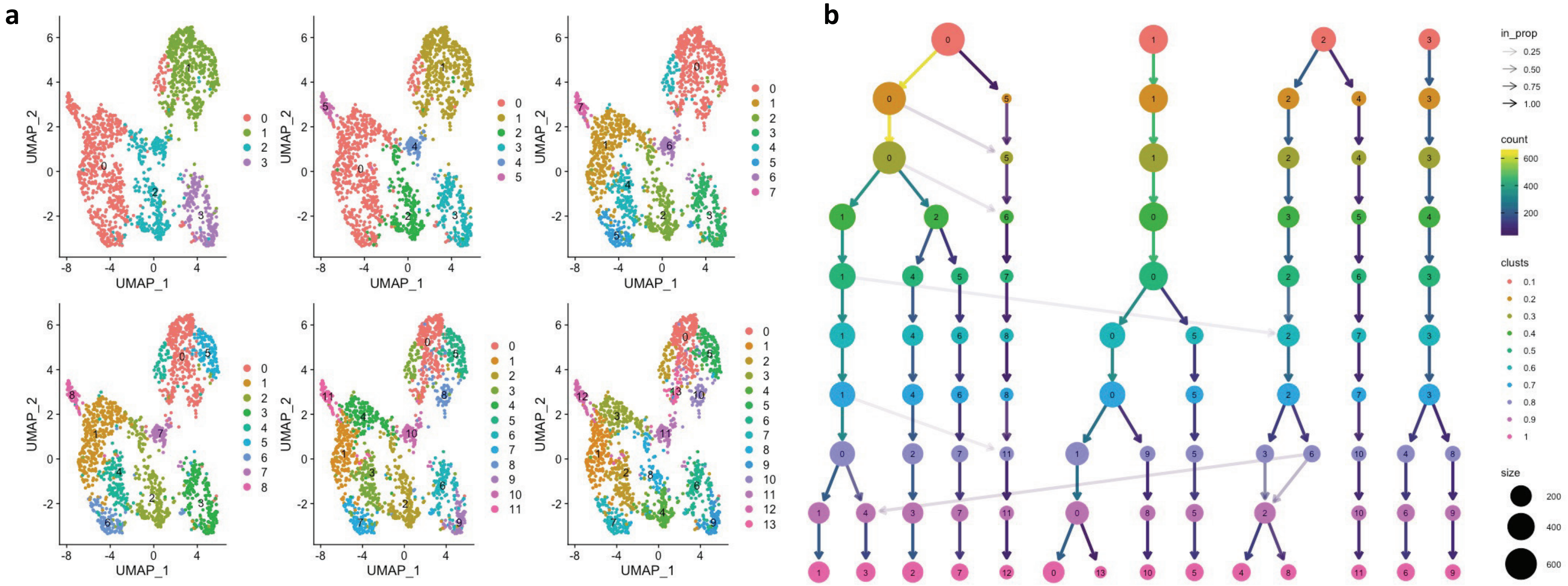

Supplementary Figure S4 – (**a**) UMAP and (**b**) ClusTree depiction of cluster identity at increasing resolutions. Related to Figure 2.

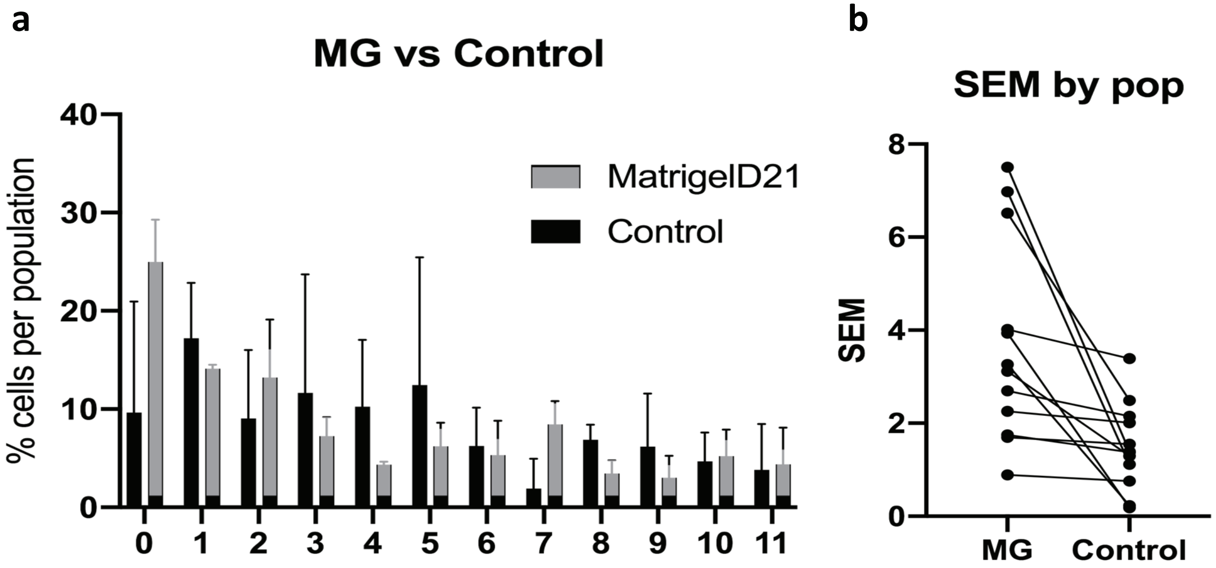

Supplementary Figure S5 – (a) Organoid composition by percentage, pooled across treatment pools. Population composition shown by percentage, error bars depict mean/SEM. Population 0 comprised 9.67 ± 6.52 vs 24.98 ± 2.49 % of the total organoid composition, in encapsulated vs control, respectively. Population 1 (17.21 ± 5.65/14.12 ± 0.23%), Population 2 (9.07 ± 6.96/13.23 ± 3.39%), Population 3 (11.64 ± 12.09/7.25 ± 1.12 %), Population 4 (10.24 ± 6.82/4.34 ± 0.18 %), Population 5 (12.45 ± 12.99/6.22 ± 1.39%), Population 6 (6.24 ± 3.91/5.32 ± 2.01 %), Population 7 (1.92 ± 3.03/8.44 ± 1.38%), Population 8 (6.89 ± 1.54/3.46 ± 0.76%), Population 9 (6.18 ± 5.40/3.01 ± 1.30%), Population 10 (4.67 ± 2.94/5.23 ± 1.55%) and Population 11 (3.83 ± 4.67/4.40 ± 2.15). **(b)** Variability across control groups is shown by expressly visualizing SEM per population. Related to Figure 2.

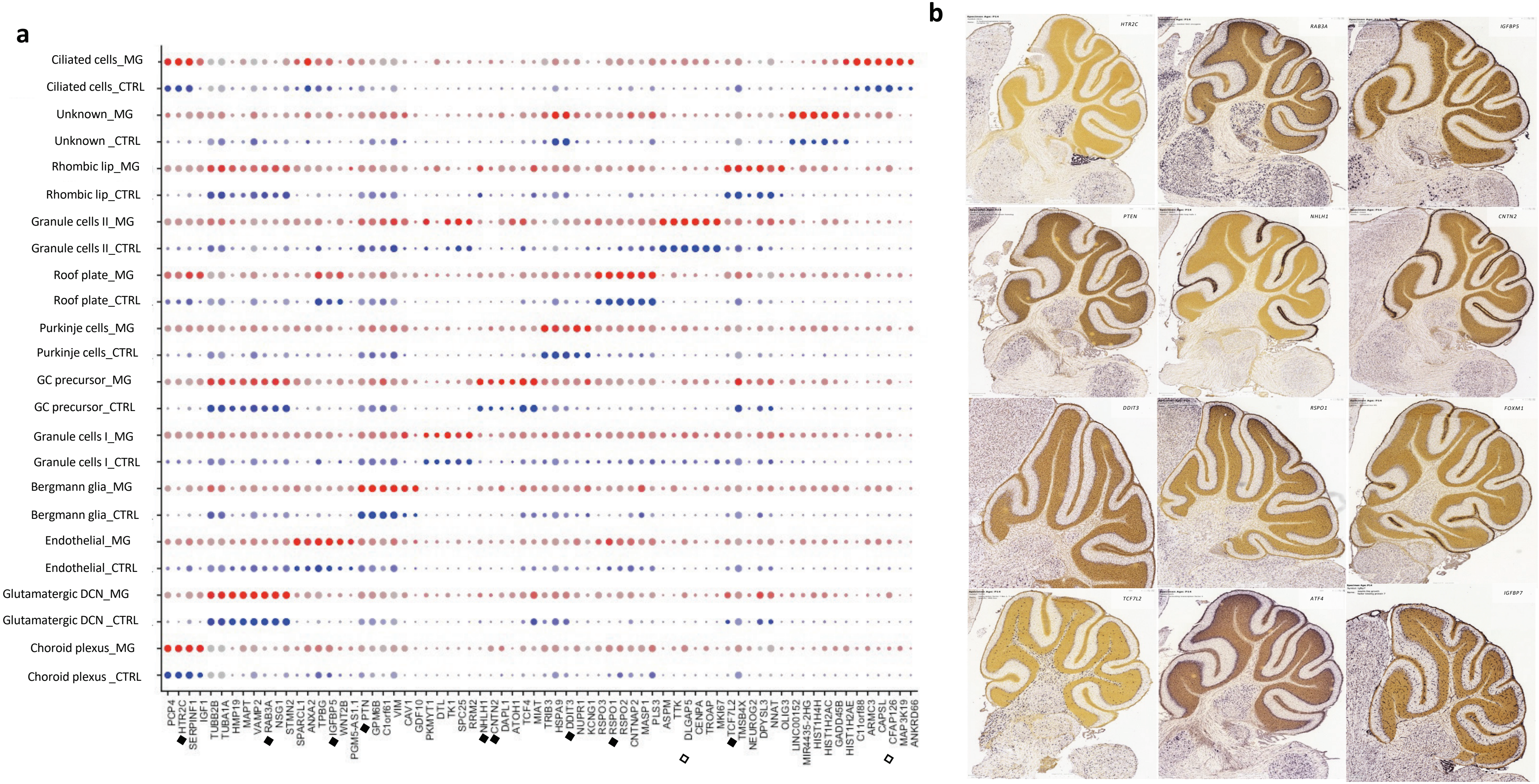

Supplementary Figure S6 – (a) Dot-plot showing expression of key markers in identified populations in Matrigel-embedded (MG) and control samples (CTRL). A solid diamond indicates marker from top 6 biomarkers was available as ISH data from the Allen Brain Atlas (ABA). Open diamond indicates sub-top6 marker. **(b)** Corresponding murine ISH sections from the Allen Brain Atlas. Related to Figure 2.

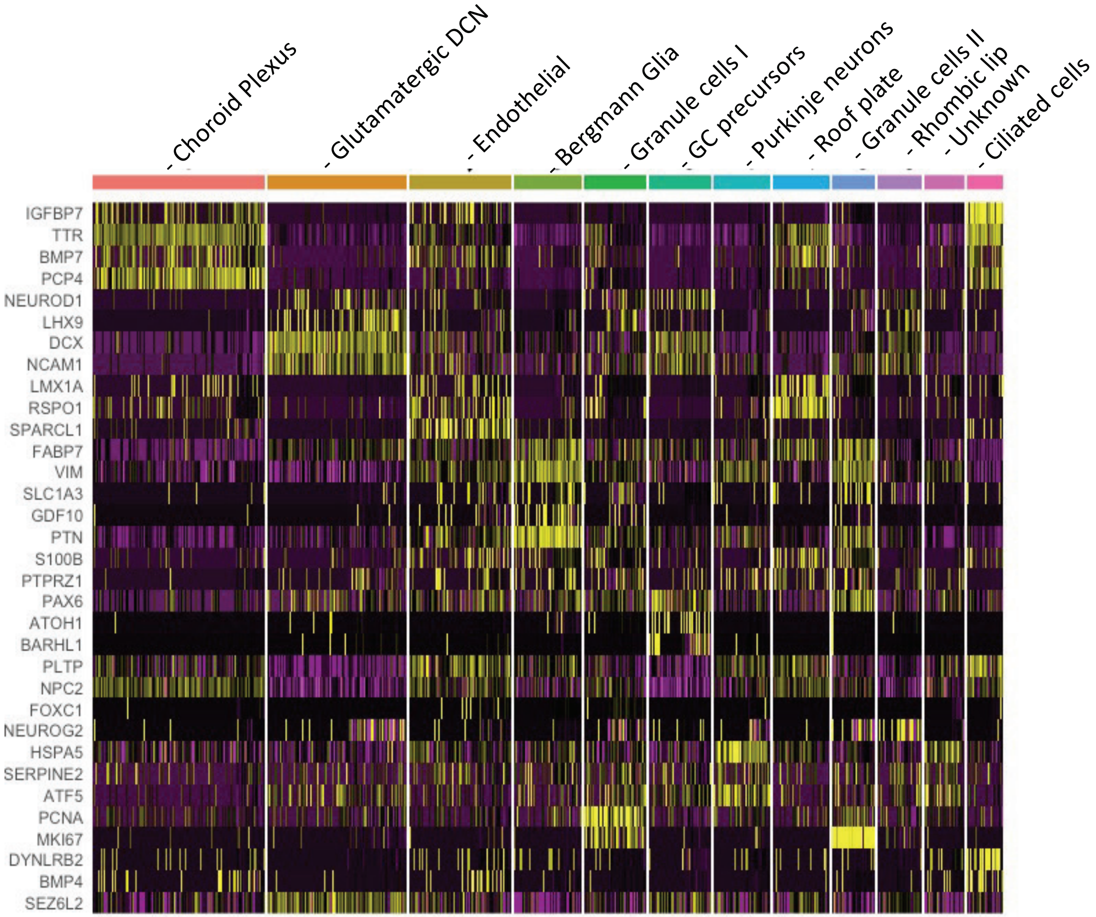

Supplementary Figure S7 – Heatmap depicting expression of canonical cerebellar cell type-specific markers in human organoids at clustering resolution 0.8. Heatmap made using Seurat (v3.2.1). Related to Figure 2.

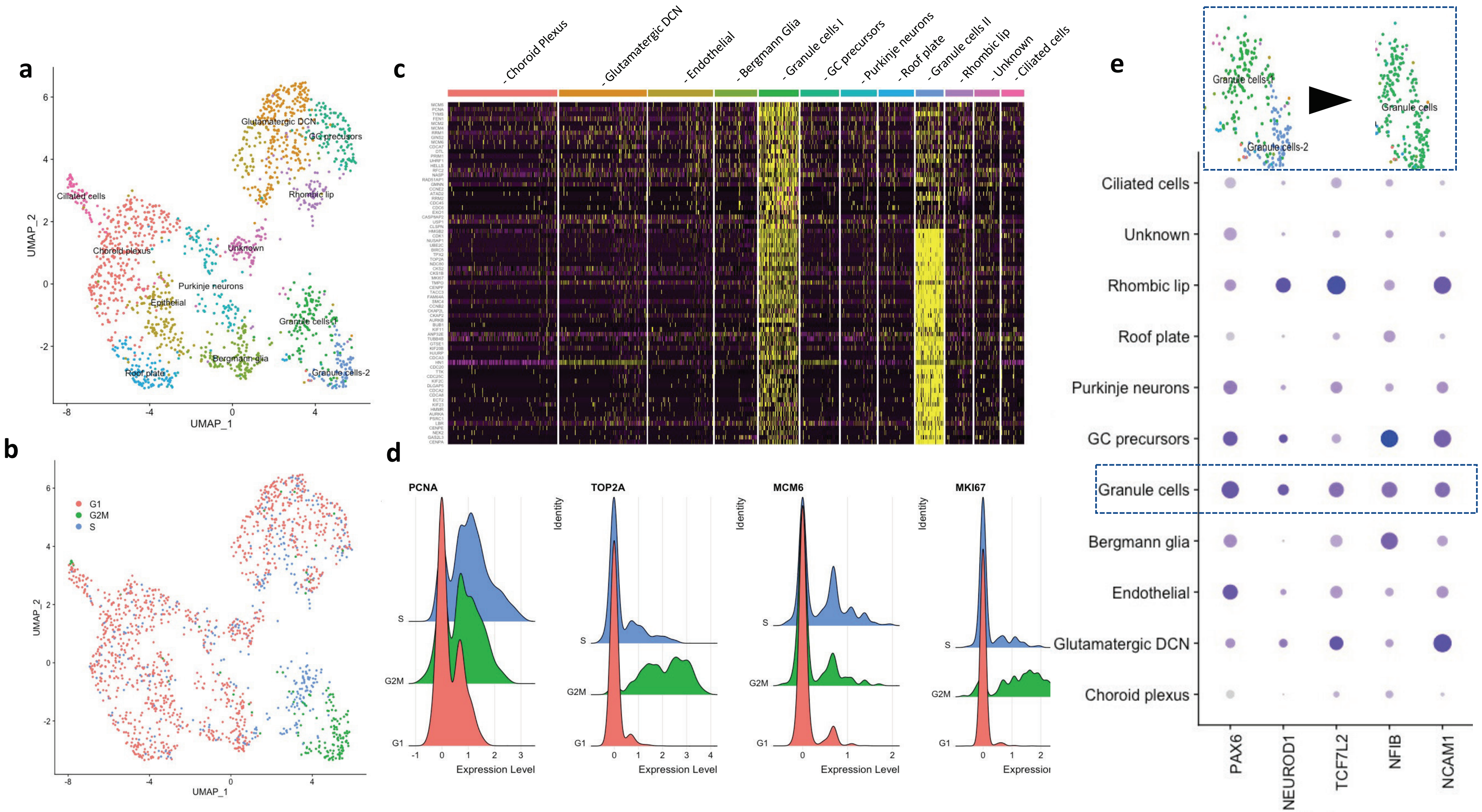

Supplementary Figure S8 – (a) UMAP projection of human organoids by cell type. **(b)** UMAP projection of human organoids coloured by predicted cell-cycle phase. **(c)** Heatmap of cell cycle-related genes. d) Ridge-plots depicting critical cell-cycle genes *PCNA, TOP2A, MCM6* and *MKI67* expression. **(e)** DotPlot showing enrichment for canonical GC genes following GC1 and GC2 cluster consolidation. Heatmap made using Seurat (v3.2.1). Related to Figure 2.

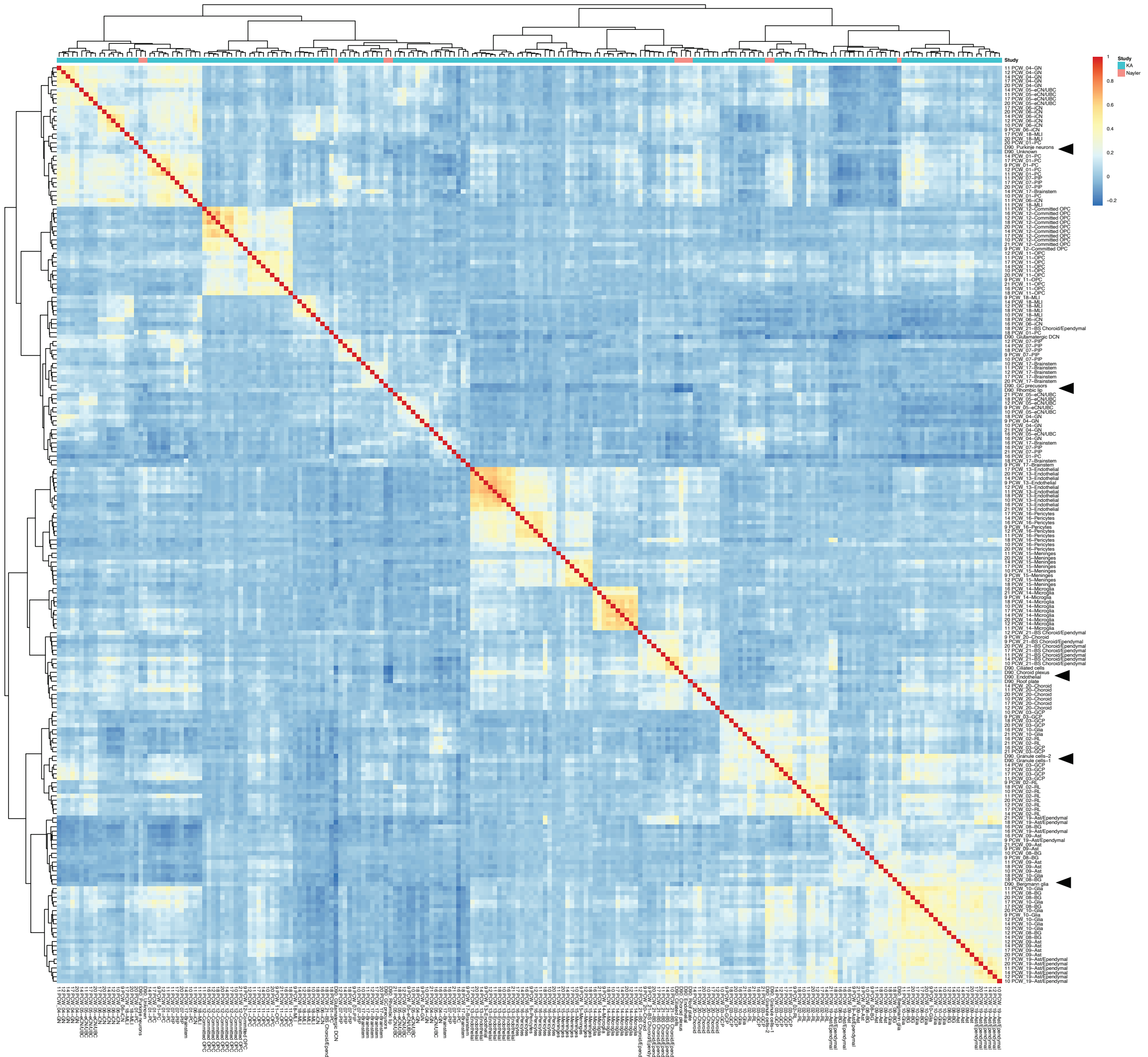

Supplementary Figure S9 – Average logged expression per cluster (cell type and time) from human cerebellar data (Aldinger et al.) against human organoids (D90), with non-parametric/CoMBat batch correction. Heatmaps were made using Pheatmap v1.0.2. Related to Figure 3.

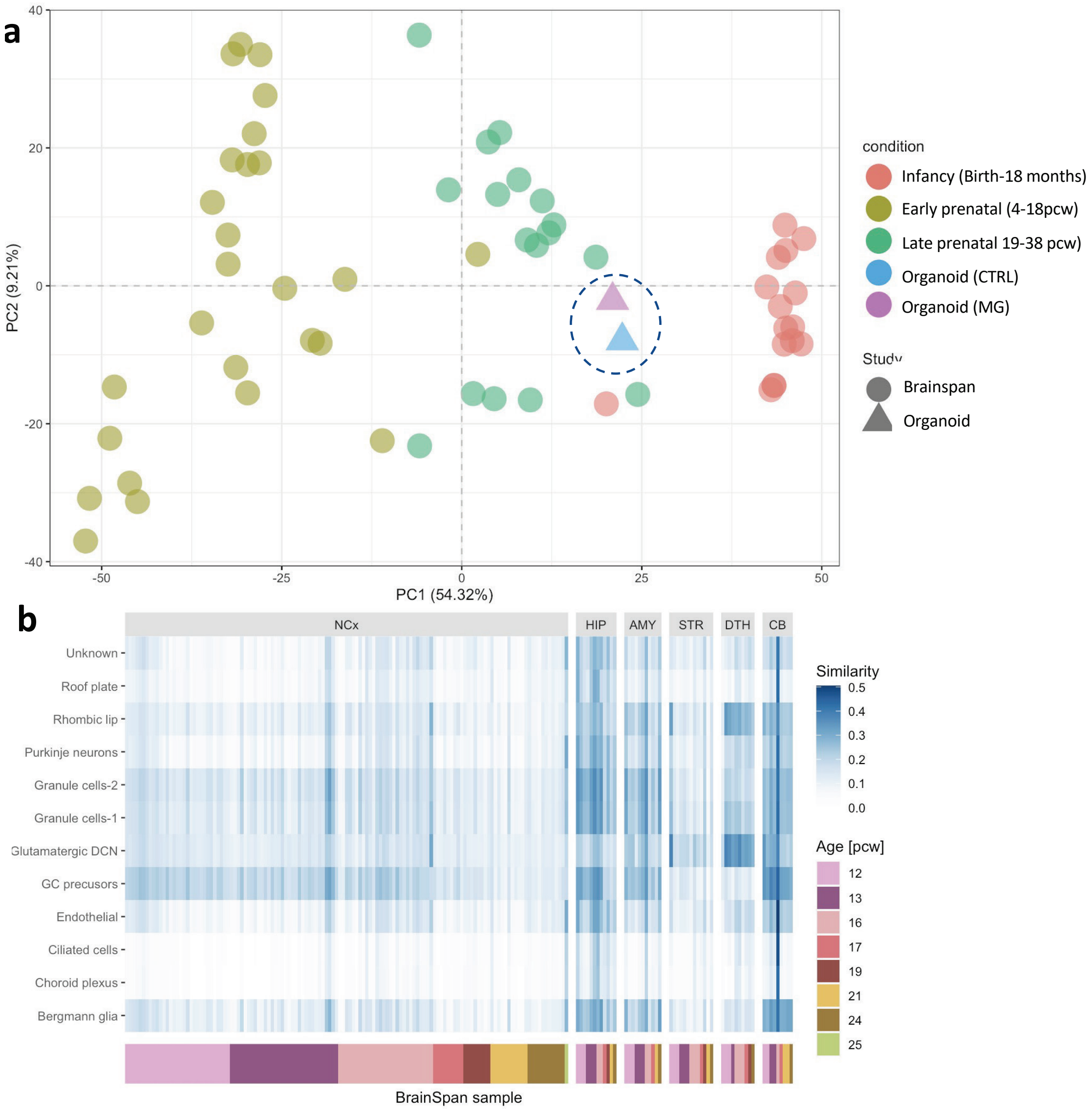

Supplementary Figure S10 – (a) PCA of human organoid and Brainspan data shows organoid samples cluster proximally to late prenatal samples. **(b)** Similarity map from Brainspan data shows time/region-specific comparisons (Top 500 DEGs, p <0.05). Related to Figure 3.

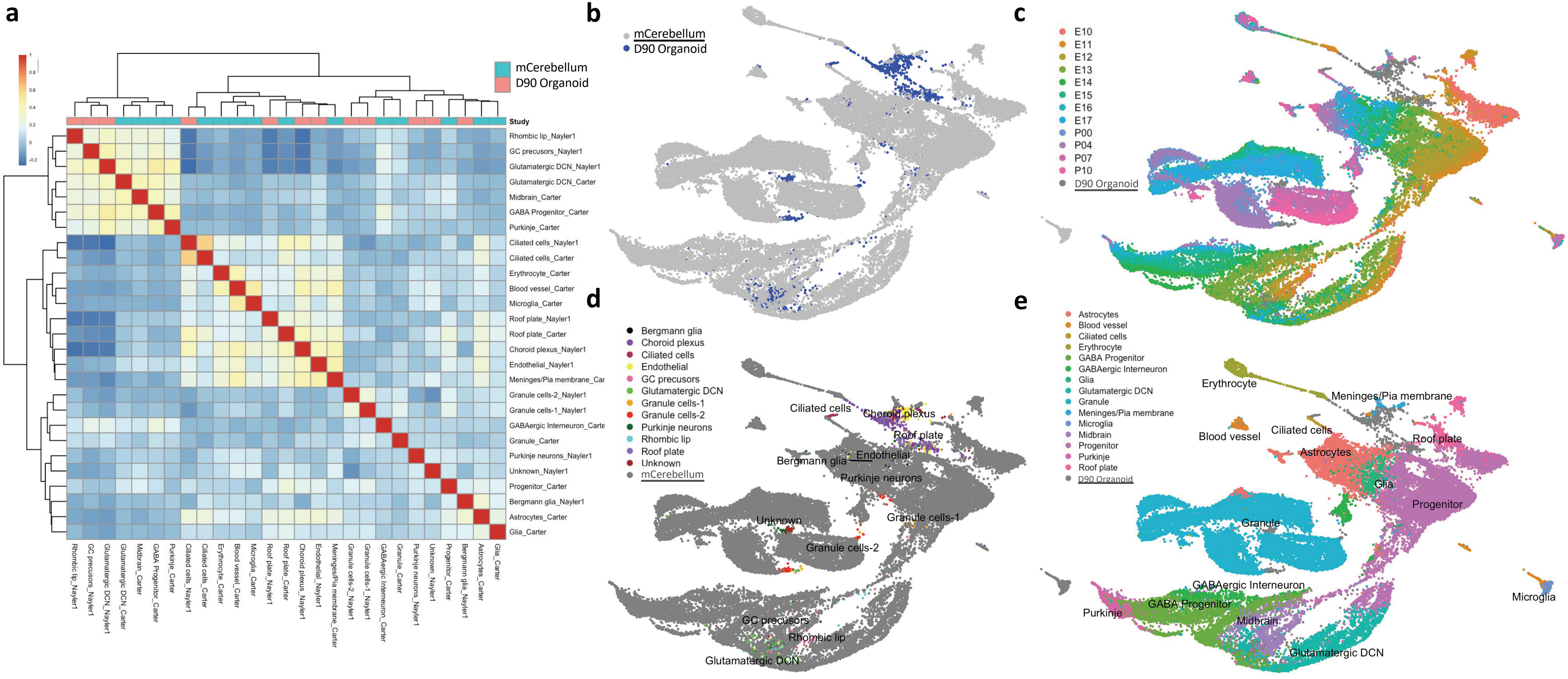

Supplementary Figure S11 – (**a**) 39,245 murine cells from 12 developmental ages were integrated with 1653 human organoid derived cells. Average logged expression per cluster (cell type) from murine cerebellar data (Carter) against human organoids (D90), with non-parametric/CoMBat batch correction. (**b**) UMAP plot of integrated, human organoid data with murine developmental data (E10-P10), grouped by cell type identity reveals close proximity of human and murine cerebellar cell types. (**c**) UMAP plot of integrated, human organoid data (grey) with murine developmental data (E10-P10, coloured) shows complex cell-type and epoch-stage clustering. Human data are represented as NA entry for visualisation purposes (grey). (**d**) Integrated object highlighting human organoid cell type identity (coloured) reveals close proximity of human organoid and murine developmental tissue cell types. Murine data are represented as NA entry (grey) for visualisation purposes. (**e**) Integrated object highlighting murine developmental tissue type identity (coloured) reveals close proximity of human organoid and murine developmental cell types. Human organoid data are represented as NA entry (grey) for visualisation purposes. The author's original cell metadata labels have been preserved for consistency. Extended clustering of pseudo-bulk cluster values are shown in Supplementary Figure S12. Heatmaps were made using Pheatmap v1.0.2.

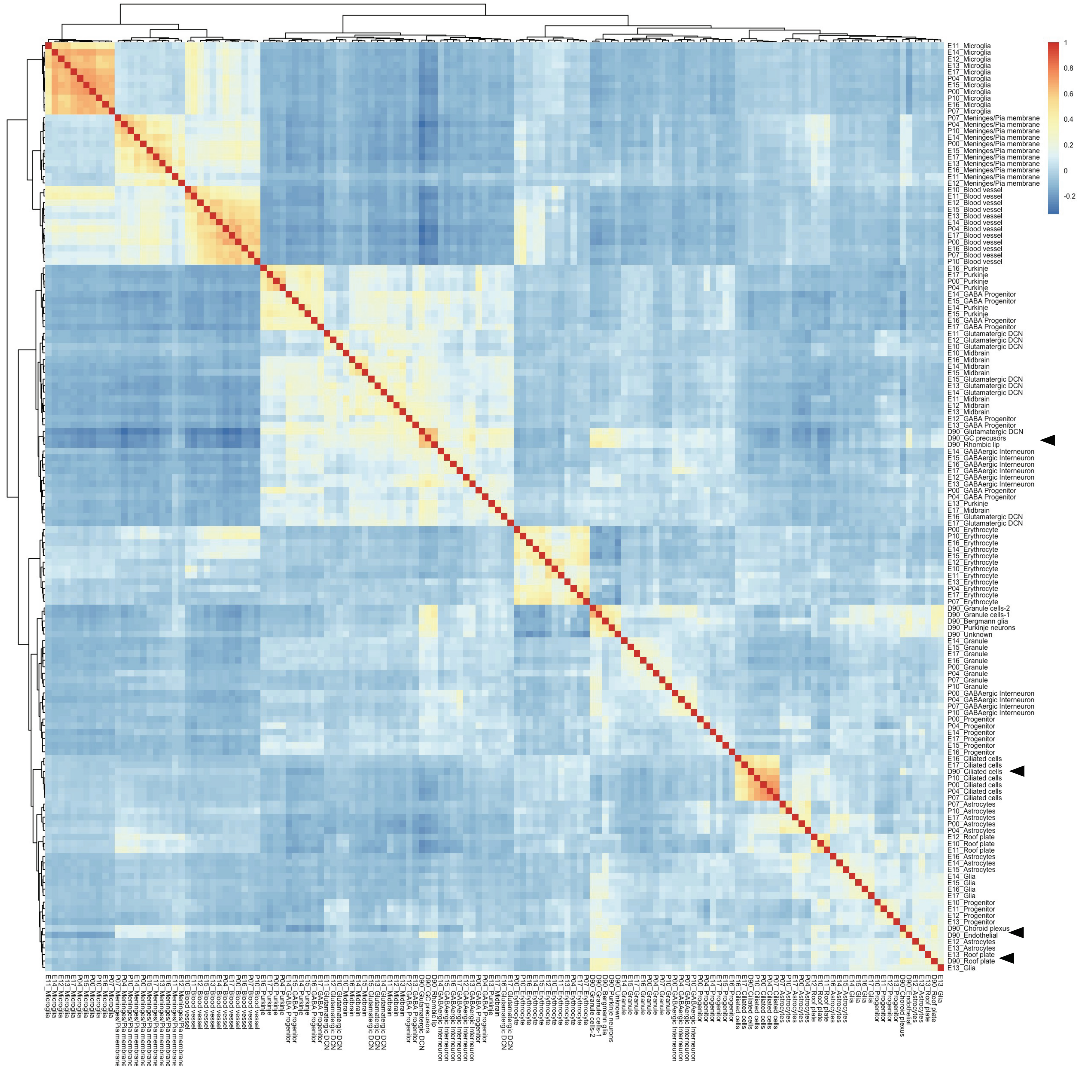

Supplementary Figure S12 – Average logged expression per cluster (cell type and time) from murine cerebellar data (Carter et al.) against human organoids (D90), with non-parametric/CoMBat batch correction. Heatmaps were made using Pheatmap v1.0.2. Related to Figure 3.

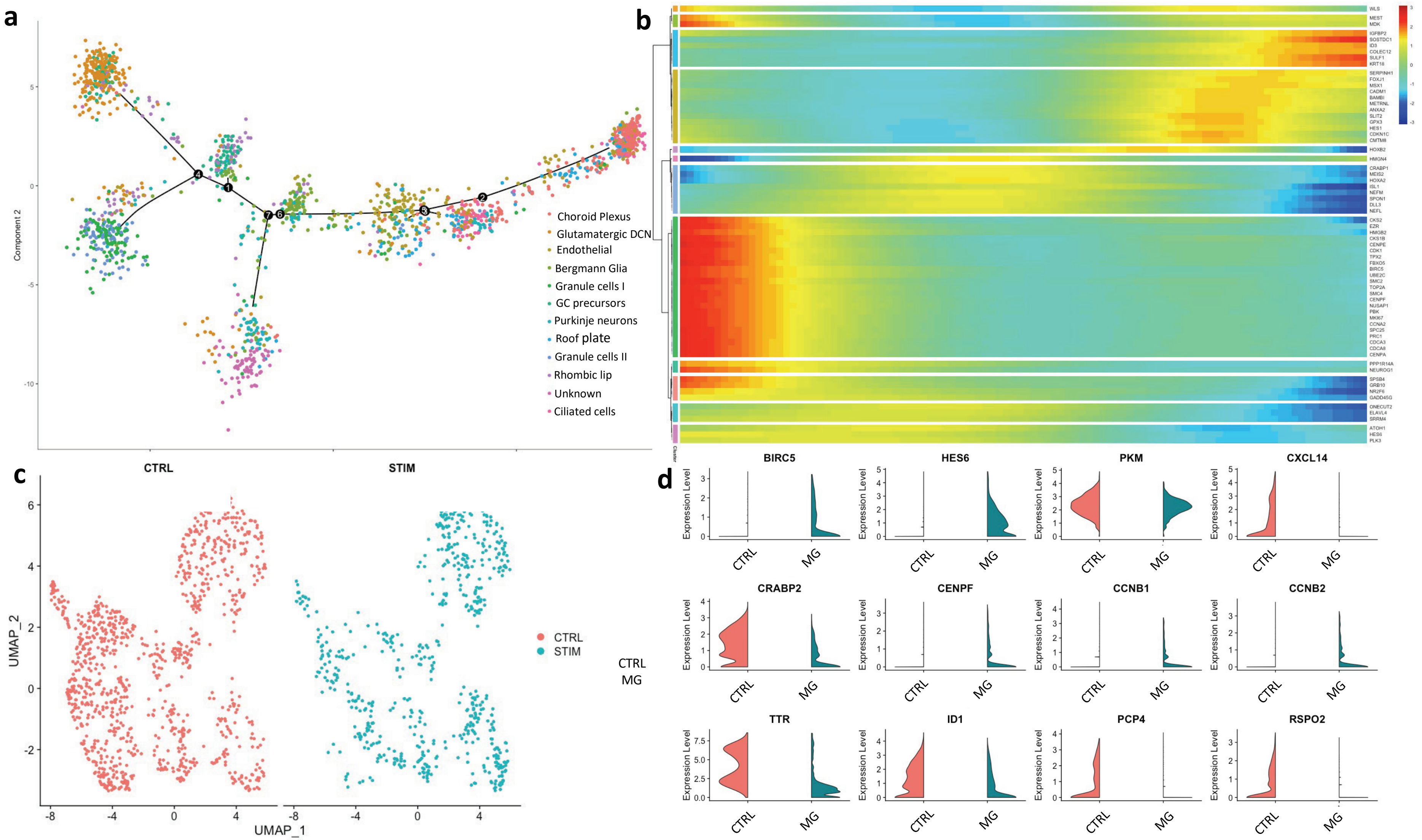

Supplementary Figure S13. Organoid composition mimics development of the cerebellum, including basement-membrane contribution to RL derivatives

(**a**) Pseudo-time reconstruction reveals a developmental trajectory akin to normal embryonic cerebellar development. (**b**) Pseudo-time plots show the contribution of canonical drivers of cerebellar specification per cell cluster. (**c**) UMAP projections of control (CTRL) and Matrigel (MG)-embedded organoids. (**d**) Top differentially-regulated genes following MG encapsulation (p-value <0.05).

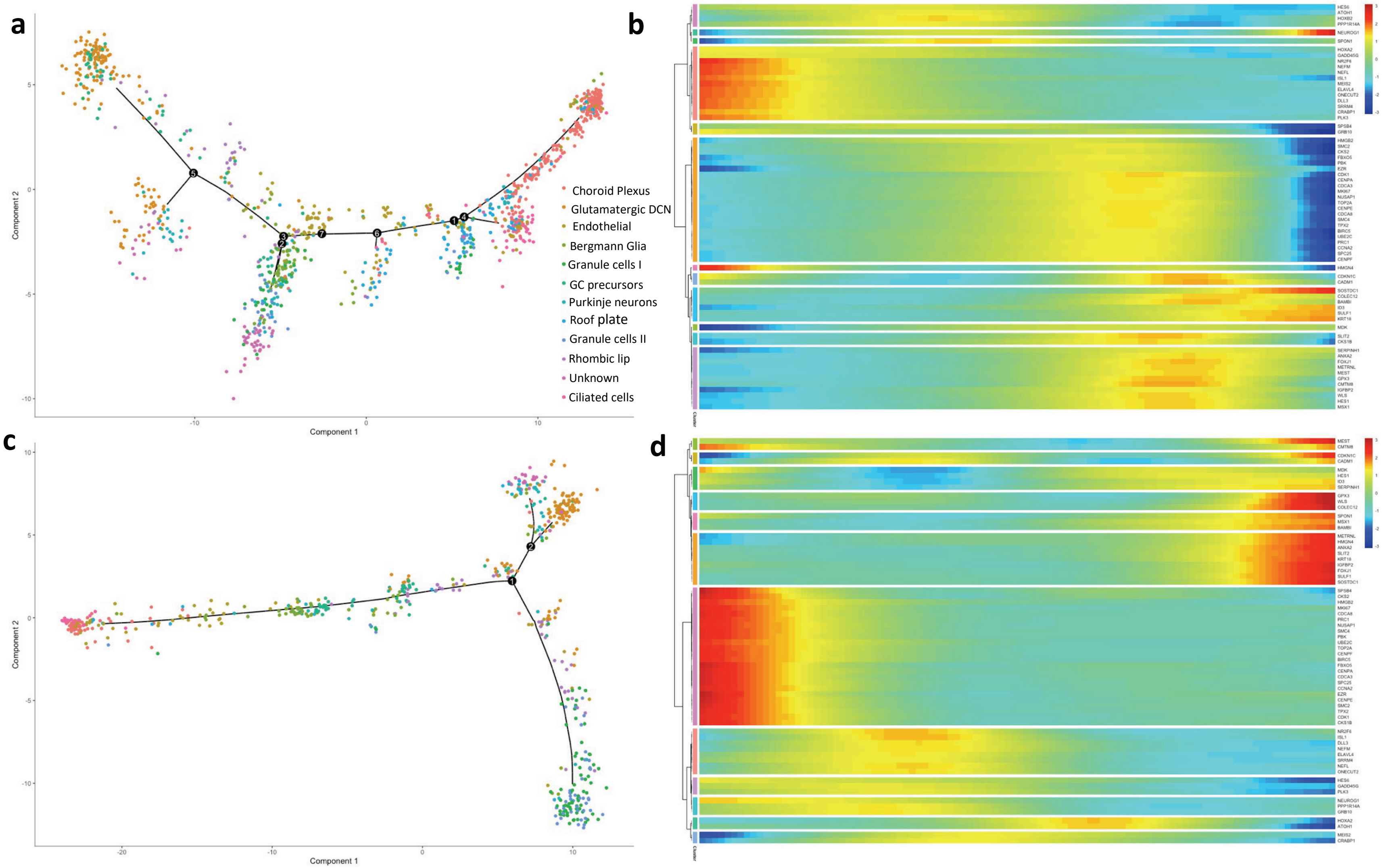

Supplementary Figure S14 – Pseudotime reconstructions of control (**a**) and Matrigel-embedded (**c**) organoids. Top genes driving branch separation are depicted in (**b**) and (**d**). Related to Figure 4.

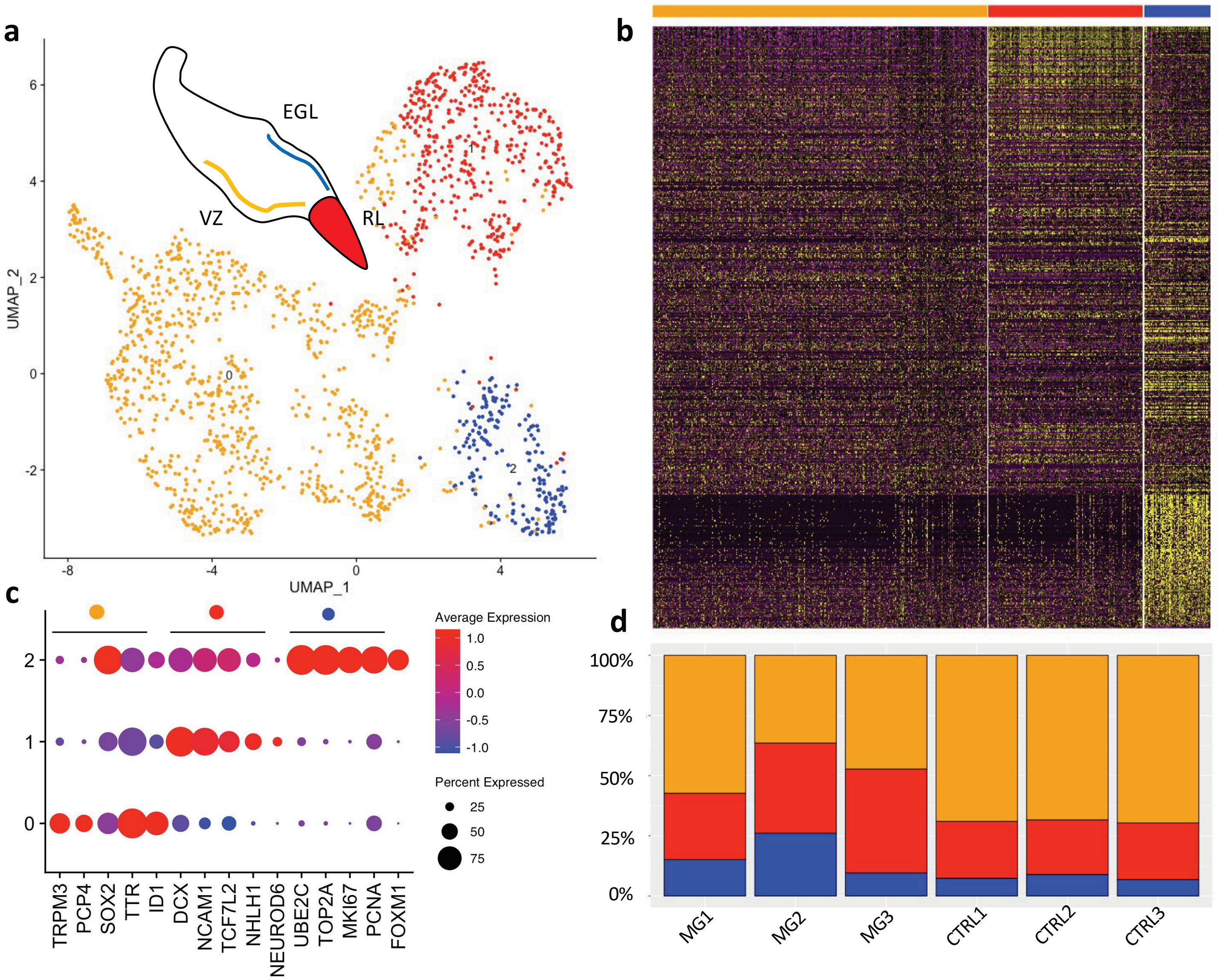

Supplementary Figure S15 – (**a**) UMAP representation of clustering to resolve GABAergic/glutamatergic lineages (VZ vs RL/EGL). (**b**) Heatmap of DE genes (Supplementary Table 16 contains full list). Heatmap made using Seurat (v3.2.1). (**c**) Dot-plot depicting key markers driving separation of clusters. (**d**) Bar graphs showing lineage-specification bias in Matrigel (teal) and Control (pink) organoids. Related to Figure 4.

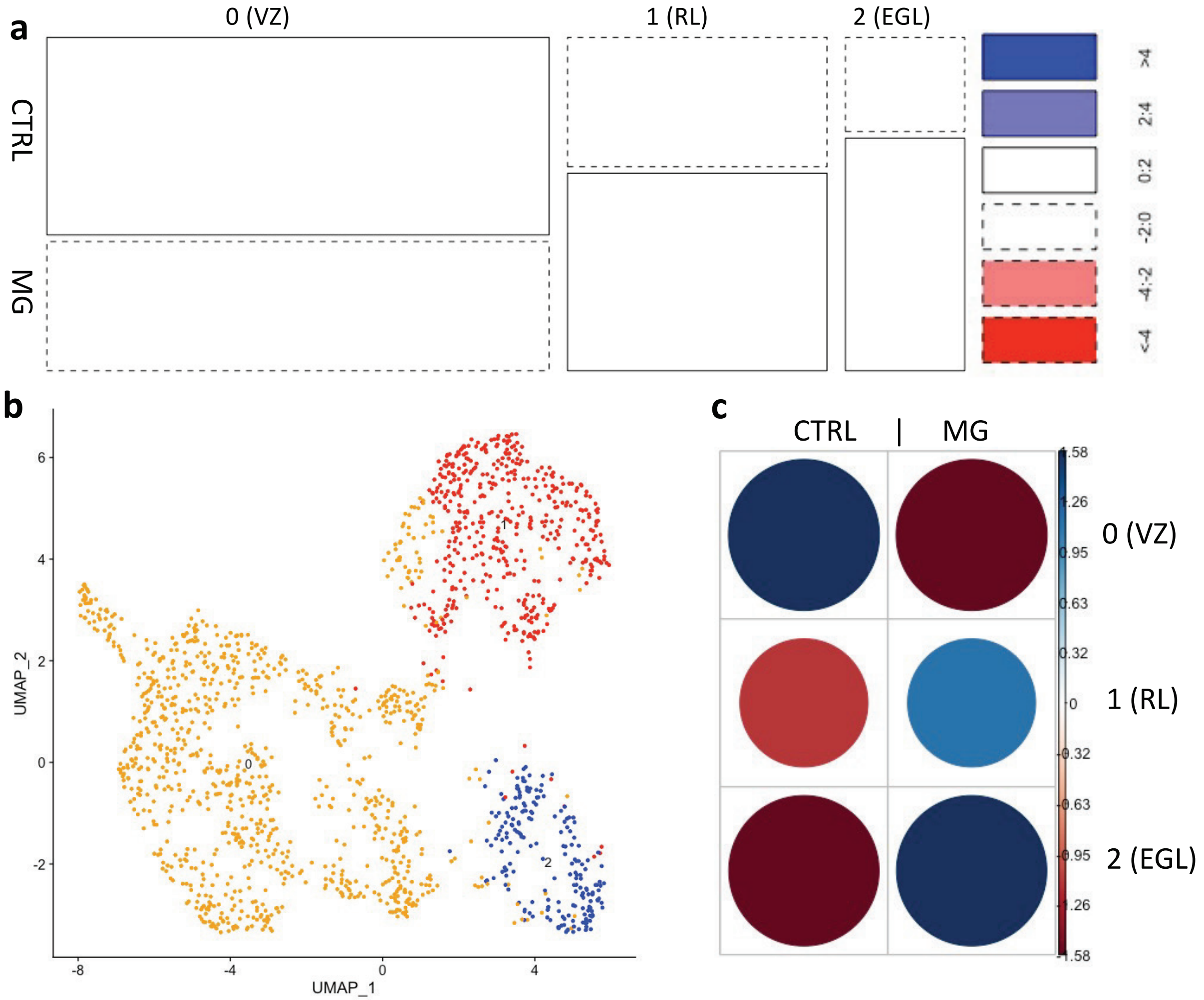

Supplementary Figure S16 – (**a**) Contingency tables for chisquare tests. Standardized residuals shown for organoid population composition clustered at a resolution to resolve GABAergic/Glutamatergic lineages (VZ vs RL/EGL). (**b**) Corresponding UMAP (**c**) Pearson standardized residuals for corresponding clustering level. Related to Figure 4.

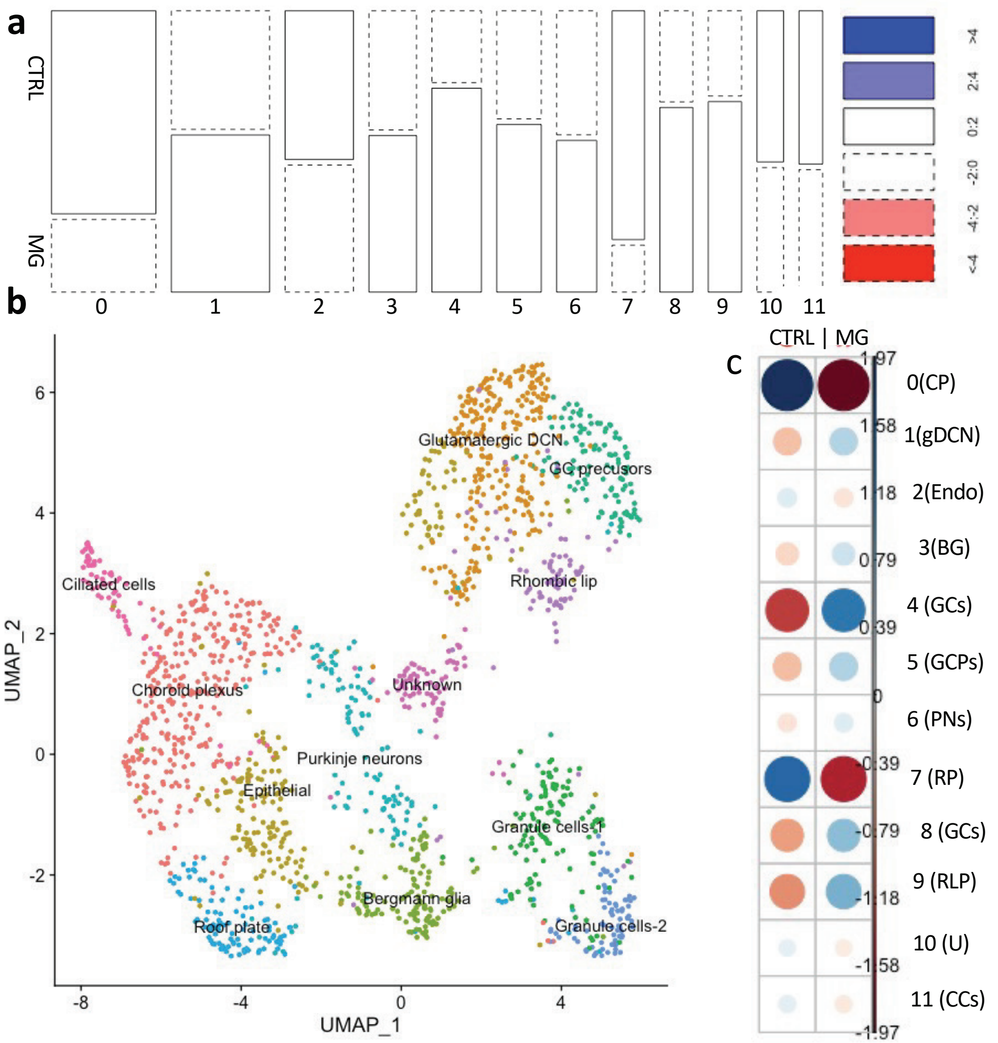

Supplementary Figure S17 – (**a**) Contingency tables for chi-squared tests. Standardized residuals for organoid population composition clustered at a resolution to resolve cerebellar populations. (**b**) Corresponding UMAP. (**c**) Pearson standardized residuals for corresponding clustering level. Related to Figure 4.

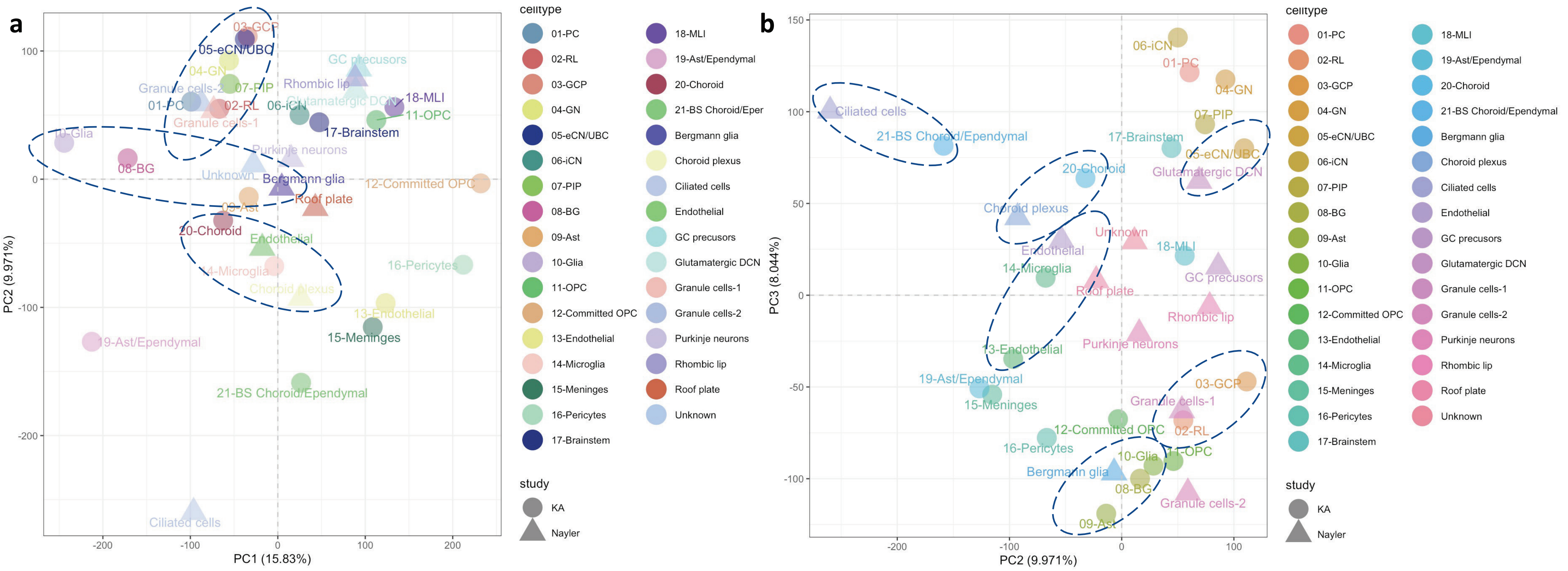

Supplementary Figure S18 – (**a**) PCA, components 1 and 2 (**b**) components 2 & 3. Dotted lines demarcate corresponding/related cell types.

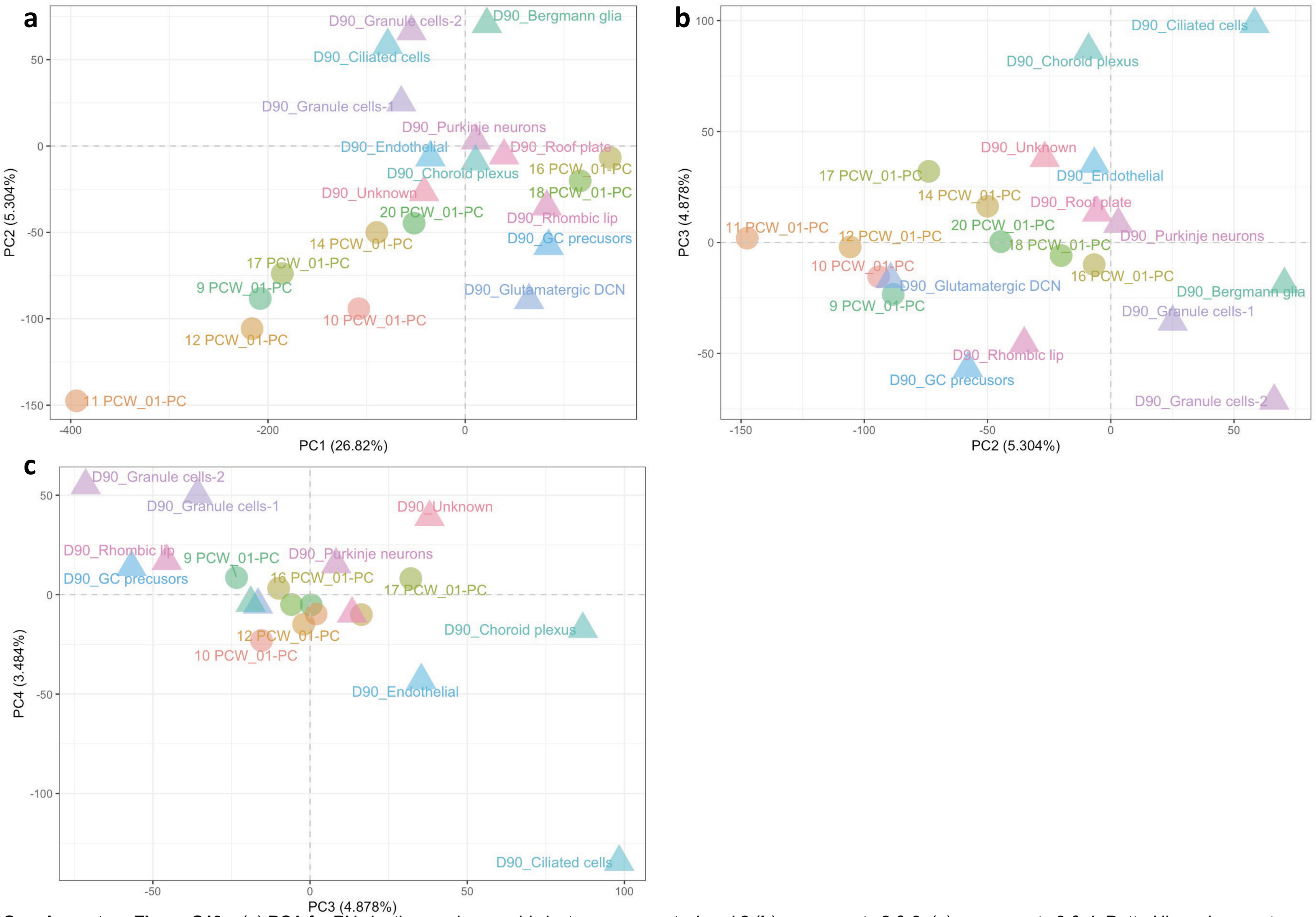

Supplementary Figure S19 – (**a**) PCA for PNs by time and organoid cluster components 1 and 2 (**b**) components 2 & 3, (**c**) components 3 & 4. Dotted lines demarcate corresponding/related cell types.

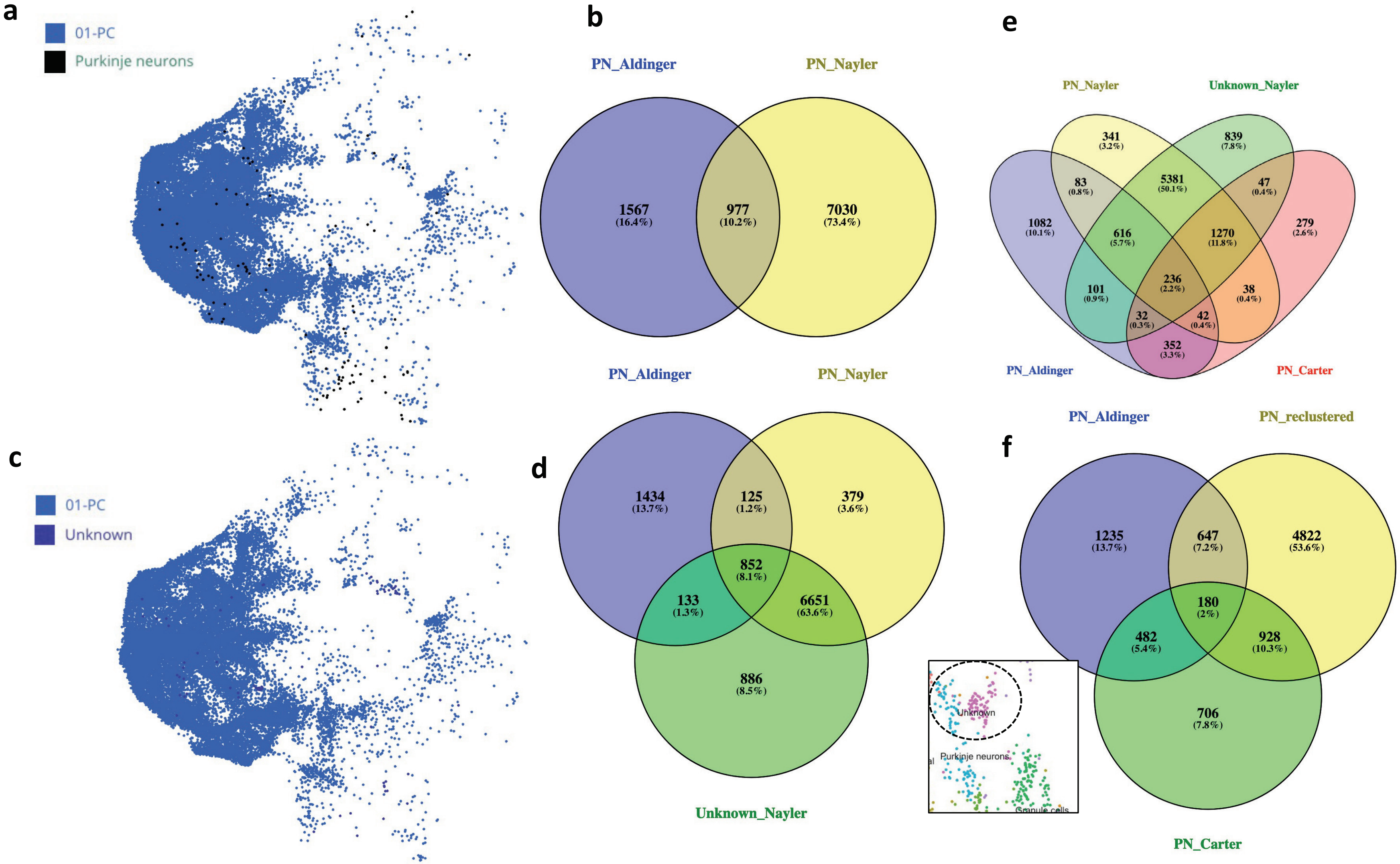

Supplementary Figure S20 – (**a**) Integrated UMAP clustering of human tissue data (Aldinger et al.) and human organoid clusters. Human tissue PCs (Purkinje cells) and organoid-derived PNs only are shown to overlap in mutual broad and diffuse space. (**b**) Integrated UMAP showing co-clustering of organoid-derived Unknown cluster shows broad and diffuse overlap with human tissue PN clusters. (**c**) Venn diagram illustrating overlap of biomarkers in human tissue PNs and organoid-derived PNs. (**d**) Venn diagram illustrating overlap of biomarkers in human tissue PNs, organoid-derived PNs and 'Unknown'. (**e**) Venn diagram illustrating overlap of biomarkers in human tissue PNs, murine PNs and organoid-derived PNs/Unknown population. (**f**) Venn diagram illustrating overlap of biomarkers in human tissue PNs, reclustered PN/Unknown & murine PNs from Carter et al.

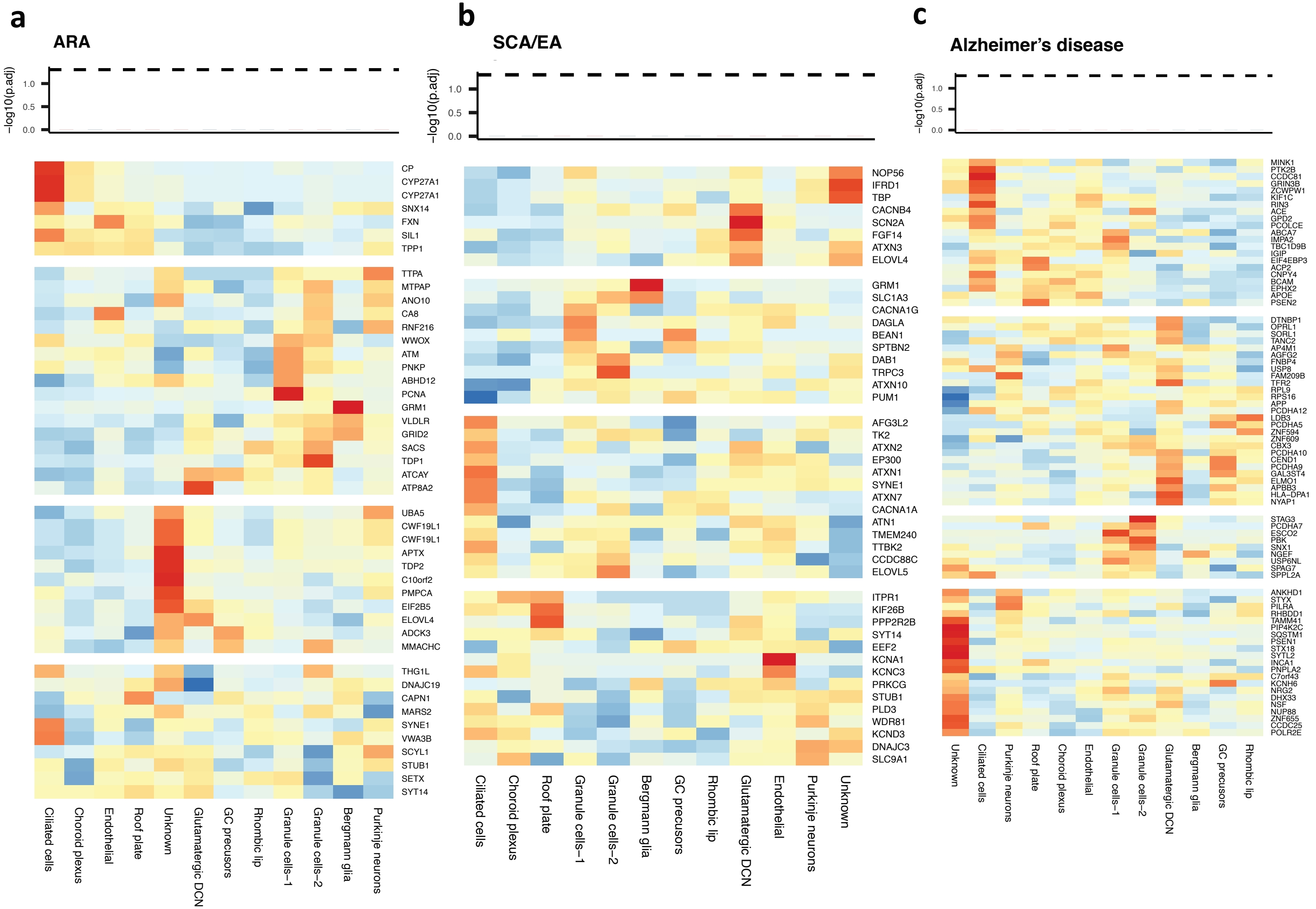

Supplementary Figure S21– Heatmaps of mean expression per organoid cell type cluster for genes associated with (**a**) Autosomal recessive ataxia, (**b**) Spinocerebellar Ataxia/Episodic ataxia and (**c**) Alzheimer's disease. Colour scheme indicates Z-score distribution. Each row represents a risk gene. Full details are included in Supplementary Table S33. Heatmaps were made using Pheatmap v1.0.2.
